# Rapamycin persistently improves cardiac function in aged, male and female mice, even following cessation of treatment

**DOI:** 10.1111/acel.13086

**Published:** 2019-12-10

**Authors:** Ellen Quarles, Nathan Basisty, Ying Ann Chiao, Gennifer Merrihew, Haiwei Gu, Mariya T. Sweetwyne, Jeanne Fredrickson, Ngoc‐Han Nguyen, Maria Razumova, Kristina Kooiker, Farid Moussavi‐Harami, Michael Regnier, Christopher Quarles, Michael MacCoss, Peter S. Rabinovitch

**Affiliations:** ^1^ Department of Pathology University of Washington Seattle WA USA; ^2^ Department of Genome Sciences University of Washington Seattle WA USA; ^3^ Department of Anesthesiology and Pain Medicine University of Washington Seattle WA USA; ^4^ Department of Bioengineering University of Washington Seattle WA USA; ^5^ Division of Cardiology Department of Medicine University of Washington Seattle WA USA; ^6^ School of Information University of Michigan Ann Arbor MI USA; ^7^ Present address: University of Michigan Ann Arbor MI USA; ^8^ Present address: Buck Institute of Aging Novato CA USA; ^9^ Present address: Oklahoma Medical Research Foundation Oklahoma City OK USA

**Keywords:** aging, echocardiography, heart, persistence, proteomics, rapamycin

## Abstract

Even in healthy aging, cardiac morbidity and mortality increase with age in both mice and humans. These effects include a decline in diastolic function, left ventricular hypertrophy, metabolic substrate shifts, and alterations in the cardiac proteome. Previous work from our laboratory indicated that short‐term (10‐week) treatment with rapamycin, an mTORC1 inhibitor, improved measures of these age‐related changes. In this report, we demonstrate that the rapamycin‐dependent improvement of diastolic function is highly persistent, while decreases in both cardiac hypertrophy and passive stiffness are substantially persistent 8 weeks after cessation of an 8‐week treatment of rapamycin in both male and female 22‐ to 24‐month‐old C57BL/6NIA mice. The proteomic and metabolomic abundance changes that occur after 8 weeks of rapamycin treatment have varying persistence after 8 further weeks without the drug. However, rapamycin did lead to a persistent increase in abundance of electron transport chain (ETC) complex components, most of which belonged to Complex I. Although ETC protein abundance and Complex I activity were each differentially affected in males and females, the ratio of Complex I activity to Complex I protein abundance was equally and persistently reduced after rapamycin treatment in both sexes. Thus, rapamycin treatment in the aged mice persistently improved diastolic function and myocardial stiffness, persistently altered the cardiac proteome in the absence of persistent metabolic changes, and led to persistent alterations in mitochondrial respiratory chain activity. These observations suggest that an optimal translational regimen for rapamycin therapy that promotes enhancement of healthspan may involve intermittent short‐term treatments.

## Graphical abstract

Cardiac morbidity and mortality increase with age in both mice and humans. Here, we demonstrate that 8‐week treatment with rapamycin is sufficient to persistently improve diastolic function, decrease age‐related cardiac hypertrophy and passive stiffness, and modulate the cardiac proteome, even a further 8 weeks after cessation of treatment. These observations suggest that an optimal translational regimen for rapamycin therapy that promotes enhancement of healthspan may involve intermittent short‐term treatments.

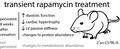

## 1 INTRODUCTION

It is estimated that by 2030, over 8 million people will suffer from heart failure (HF) in the United States alone (Heidenreich et al., 2013). In the developed world, HF is the condition most responsible for poor healthspan in males (age‐standardized years lived with disability; Moran et al., 2014). The estimated cost of HF in the United States is estimated to be $70 billion by 2030 (Heidenreich et al., 2013). Historically, most attention has been focused on HF with reduced ejection fraction (HFrEF), such as may result after myocardial infarction; however, the Atherosclerosis Risk in Communities study reported that 47% of US hospitalizations due to HF were due to HF with preserved ejection fraction (HFpEF; Chang et al., 2014). HFpEF is generally defined clinically by signs and/or symptoms of HF combined with preserved left ventricular (LV) ejection fraction (EF). In this setting, reduced cardiac output is related to impaired diastolic LV filling, and this results in exercise intolerance and contributes to frailty. Cardiac aging in both humans and mice is characterized by a progressive decrease in diastolic function and an increase in LV hypertrophy (Chiao & Rabinovitch, 2015). These similarities make aging rodents a good model for study of pharmacotherapies directed toward correcting diastolic function (Dai & Rabinovitch, 2009). While in recent decades medical management has enjoyed substantial success in improving health and survival of patients with HFrEF, effective treatment for HFpEF has been elusive. Despite the efforts of several large randomized clinical trials designed to improve quality of life in patients with HFpEF, results have thus far been largely disappointing (Plitt, Spring, Moulton, & Agrawal, 2018).

Rapamycin is an FDA‐approved drug that directly inhibits the mechanistic target of rapamycin (mTOR) Complex I (C1). Inhibition of mTORC1 has wide‐ranging effects in vivo, including altering protein synthesis, inhibiting cell growth, and stimulating stress response mechanisms and autophagy. Transient or lifelong treatment extends lifespan and/or healthspan in many organisms, ranging from nematodes to primates (Bitto, Wang, Bennett, & Kaeberlein, 2015). Rapamycin extends murine lifespan in both sexes, even when administered at 9 or 20 months of age in genetically heterogeneous mice (Harrison et al., 2009; Miller et al., 2011), and in C57BL/6 mice at 19 (Zhang et al., 2014) or 20–21 months of age (Bitto et al., 2016). The lifespan and healthspan extension due to rapamycin is both dose‐ and sex‐dependent (Miller et al., 2014). Clinically, rapamycin and so‐called “rapalogs” are used to prevent rejection after organ transplantation (Fine & Kushwaha, 2016) and for the prevention of restenosis after insertion of cardiac stents (Park et al., 2013). Major concerns in considering potential clinical translation of rapamycin treatment are potential detrimental effects that include immunomodulation, gonadal atrophy, and stomatitis (Boers‐Doets et al., 2013; Pallet & Legendre, 2013). However, these adverse effects are generally reversible, leading to the question of whether the more desirable healthspan effects of rapamycin might persist after the undesirable effects have resolved.

Work from our laboratory and others has shown that continuous rapamycin improves cardiac function, most specifically diastolic function, when administered to middle‐ or late‐aged mice (Dai et al., 2014; Flynn et al., 2013). Rapamycin can also improve cardiac structure and function in the context of various genetic and experimental conditions that promote cardiac disease (Das et al., 2014; Paul et al., 2014).

In this study, we analyzed functional and molecular outcomes from continuous and transient rapamycin treatment in aged, male and female C57BL/6 mice. In both sexes, rapamycin treatment replicated our previous results showing a significant improvement in cardiac diastolic function, and this effect was persistent for 2 months after rapamycin was eliminated from the diet. By focusing on molecular changes due to rapamycin treatment that persist after drug removal, we hoped to shed light on the specific mechanisms of cardiac functional rejuvenation induced by rapamycin treatment.

## 2 RESULTS

### 2.1 Rapamycin persistently improves diastolic function

**Figure 1:**
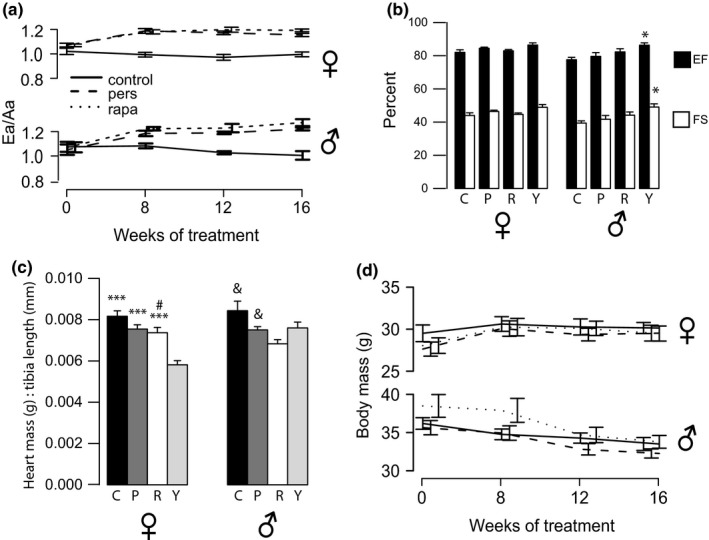
Rapamycin persistently improves diastolic function and reverses cardiac hypertrophy. (a) Ea/Aa ratios of female and male mice over the course of treatment (average ± *SEM*). Continuous rapamycin treatment (rapa, dotted line), persistence (pers, dashed line), and aged control (control, solid line). Both rapa and persistence groups are statistically significantly higher than controls for weeks 8, 12, and 16 by one‐way ANOVA followed by Tukey's post hoc for all groups at each time point per sex. Rapa and persistence groups’ Ea/Aa increased significantly by one‐way ANOVA with repeated measures for each group over time. (b) Systolic function parameters measured by echocardiography at 16 weeks. EF: ejection fraction; FS: fractional shortening. Black bars, %EF; white bars, %FS; C = control, P = persistence, R = rapa, Y = young. *significant by *t* test between old control and young groups, †significant by *t* test between pers and young groups. (c) Heart mass in grams normalized to tibia length (mm) for all groups at 16 weeks. *p*‐values from Tukey's post hoc tests when sex‐specific one‐way ANOVA was significant. * versus young, # versus control, and versus rapa. (d) Body mass in grams for all groups over time. *p < .05, **p < .01, ***p < .001

In both humans and mice, diastolic function is measured by comparing the relative proportion of LV filling that takes place in early diastole by LV relaxation (Ea) versus that takes place in late diastole, secondary to atrial contraction (Aa). In healthy hearts, the early component is greater than the late component, and diastolic dysfunction is conventionally ascribed when there is a reversal of this ratio, that is, an early‐to‐late filling ratio below 1.0. At the start of the study, the 24‐month‐old male and 22‐month‐old female mice (both at 75th percentile of lifespan; Turturro et al., 1999) demonstrated an Ea/Aa ratio averaging close to 1 (approximately half the mice above and half below 1.0), which is typical of this age in C57BL/6NIA mice. In the control group, this ratio stayed steady, but animals exposed to rapamycin for 8 weeks improved their diastolic function significantly (Figure 1a). As noted previously, this level of improvement brought the Ea/Aa ratio back approximately halfway to that of young mice (Chiao et al., 2016; Dai et al., 2014). After cessation of treatment, rapamycin‐induced improvement persisted for an additional 8 weeks, with cardiac performance maintained at levels near those of mice receiving 16‐week continuous rapamycin treatment (diastolic function, 82% persistent in females and 78% in males at 16 weeks).

Our previous work in 24‐ to 26‐month‐old female C57BL/6NIA mice showed no significant change in systolic functional measures (fractional shortening, FS and EF) during 10 weeks of rapamycin treatment (Dai et al., 2014). Concordantly, there were no measurable differences in FS or EF in the female mice at 16 weeks in this study (Figure 1b). However, males showed a small, but statistically significant, reduced FS and EF in old compared to young control mice. While rapamycin‐treated old mouse EF and FS were intermediate between young and old values for both sexes, these differences did not reach significance.

We quantitated cardiac hypertrophy by measuring cardiac weight normalized to tibia length at necropsy (Figure 1c). Female mice at 16 weeks showed a decrease in cardiac hypertrophy after rapamycin treatment, and this effect trended toward persistent (*p* = .086) by *t* test. Males also showed a reduction in hypertrophy with rapamycin treatment (*p* = .012), and again, this difference approached significance in the persistence group (*p* = .081) by *t* test. Combining both sexes by scaling all data to the same‐sex control average did yield a significant difference between old and persistence groups (Figure S1). The reduction in cardiac hypertrophy cannot be explained by reduction in overall body size, as the body weight over time in all groups was similar and relatively stable (Figure 1d). Young animals were smaller for both sexes (mean ± *SEM* F: 21.01 ± 0.60, M: 29.57 ± 0.39).

### 2.2 Passive stiffness of the left ventricle is decreased with rapamycin

**Figure 2:**
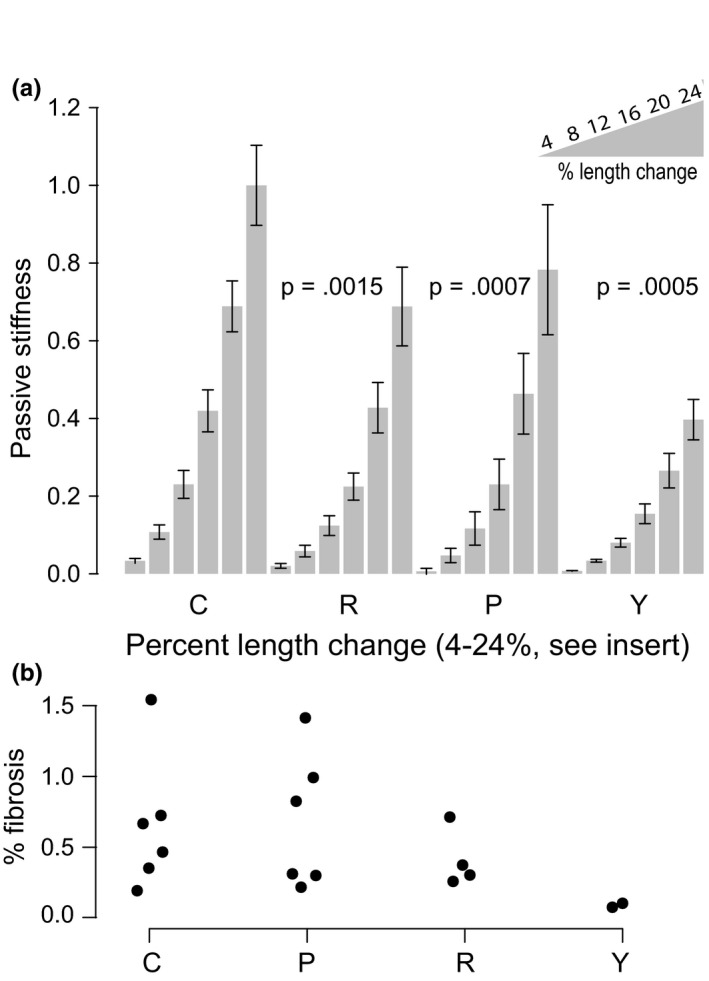
Passive stiffness increases with age and is persistently decreased with rapamycin. (a) Passive force for each mouse in each condition is shown for six length changes. All data were normalized to the control animals at 24% length change. Mean ± *SEM* is shown for each group at each length change. *p*‐values are from linear modeling of log‐transformed data. *N* = 3~12 mice per group. (b) Percent positive picrosirius red staining in ventricle slices. Each dot is median % fibrosed area for one mouse. C: control; R: rapamycin; P: persistence; Y: young

To examine whether the change in diastolic function could be due to passive rather than active relaxation of the left ventricle, we extracted LV multicellular preparations from our control, rapamycin, persistence, and young animals (*n* = 5~8 per group) and tested how much force it took to passively stretch the demembranated myocardium (Figure 2a). This stiffness generally increases with age in mice, dogs, and humans (Asif et al., 2000; Campbell & Sorrell, 2015). We found that rapamycin treatment significantly and persistently reversed the age‐related increase in passive stiffness of the preparations. We did not detect a significant difference in the passive stiffness of rapamycin‐treated groups compared to persistence groups. These data suggest that myocardial passive stiffness is a significant contributor to the diastolic dysfunction seen with aging and that rapamycin can persistently reverse this effect. One potential cause of increased passive stiffness is increased fibrosis of the extracellular matrix of the ventricular wall (Jalil et al., 1989). We examined this by staining with picrosirius red and quantifying percent of collagen deposition detected under polarizing light. We observed no significant differences between old groups (control, rapamycin, and persistence; Figure 2b).

### 2.3 Rapamycin dramatically alters protein abundance in both sexes; however, the persistence of these changes varies by sex

**Figure 3:**
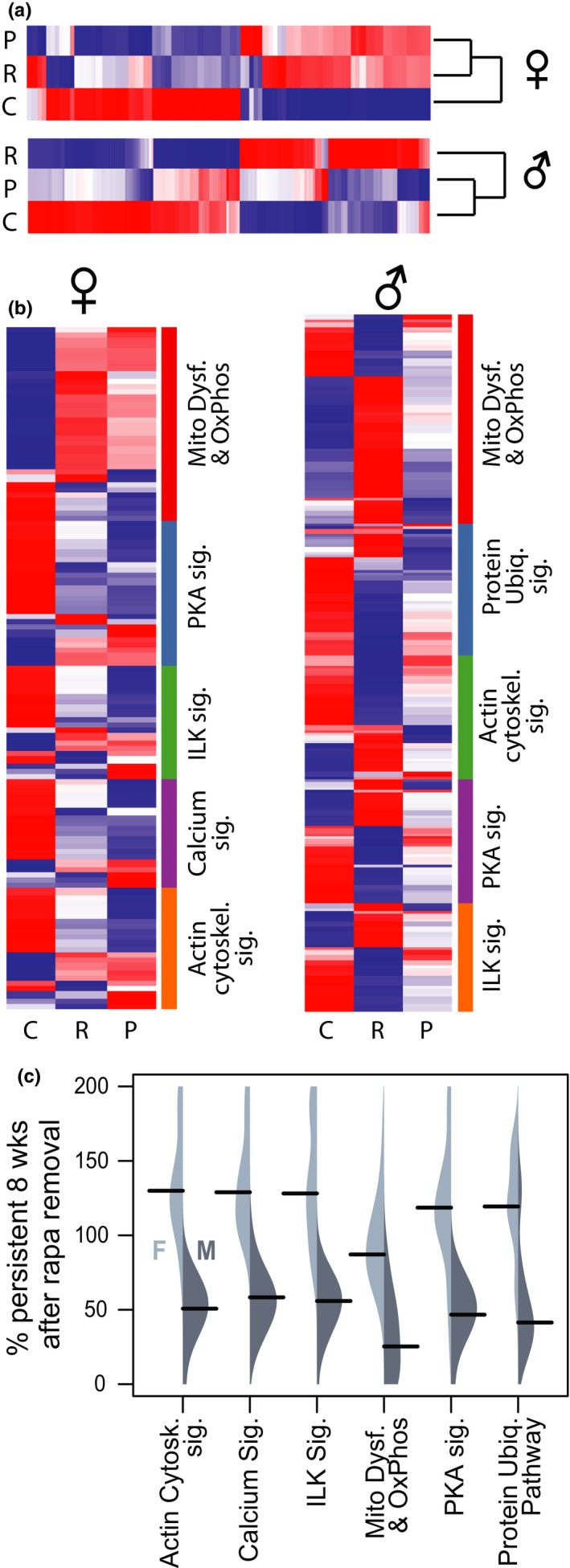
Persistence of abundance changes in proteins in top IPA pathways differs by sex. (a) Dendrograms and heatmaps showing all significantly altered protein abundances due to rapamycin for each sex. Dendrograms show Spearman's distance as a measure of relatedness. Color shows *z*‐scores of protein abundance differences by protein, with red indicating greater abundance and blue meaning less abundance (−1.15 > *z* > 1.15). (b) *Z*‐score heatmaps of protein abundance, organized into the five most significantly altered pathways (by IPA) for each sex—females on the left and males on the right. (c) Asymmetrical beanplots show the range of the percent persistence for proteins in each IPA category (*y*‐axis), with females (light gray) on the left side of each bean and males (dark gray) on the right. Black bars denote the median of the range for each sex/category. The data range was limited to 0%–200% for easier visualization. All data shown are from tissue collected at the 16‐week time point in the old mice. C—untreated control, R—continuous rapamycin treatment, and P—persistence 8 weeks after cessation of 8‐week treatment. Protein names are shown in Tables S1~S4

In our previous work, we applied proteomics to detect many differences in protein abundances due to 10‐week rapamycin treatment beginning at 24 months in female C57BL/6NIA mice (Dai et al., 2014). Thus, an important question was whether the changes in proteome abundance with rapamycin treatment were persistent after drug removal. Figure 3a shows heatmaps of the set of all proteins in each sex which had a significant difference between control and continuous rapamycin treatment at the 16‐week time point (by Student's *t* test, adjusted for multiple comparisons, as described in Methods). (Figure S2 shows principal component analysis for males and females using the same data as Figure 3a) In the comparison between continuous treatment and persistence groups, females had close similarities between these two groups, while the males showed persistence group protein abundances that were intermediate between old control and continuous rapamycin. Qiagen Ingenuity Pathway Analysis (IPA) software was used to identify significantly changed canonical pathways. This revealed that eight of the top 10 pathways were conserved between sexes. The five most significantly changed pathways in each sex are shown in the heatmap of Figure 3b, four of which are conserved between sexes; again, it is apparent that the rapamycin and persistence groups are similar in the females, but in the male cohort, the persistence group is more intermediate. Gene names and *z*‐scores for all heatmaps are listed in Tables S1–S4. The distribution of percent persistence of the proteins within each IPA category is plotted in Figure 3c. It can be seen that for all top six pathways but mitochondrial dysfunction, the median protein abundance in the female persistence groups is actually a larger change (120%–125% effect in the same direction) than in the continuous rapamycin treatment group, whereas the median mitochondrial dysfunction pathway persistence is ~86% persistent in females (Table S5). As predicted by the overall proteomics, persistence within IPA pathways was appreciably lower in males than females and differences between pathways were less apparent.

The mitochondrial dysfunction pathway is a larger set of proteins (females *n* = 27, males *n* = 55) compared to the other top five pathways (females *n* = 6–18, males *n* = 19–29), and was more heterogeneous. When electron transport chain (ETC) proteins alone were examined, persistence was varied between the sexes and the individual complexes of the ETC (Table 1), with the females again generally showing greater persistence in each complex than the males. Interestingly, the mean persistence of proteins in Complex V of the respiratory chain (ATP synthase) was very high in both sexes. Many of the proteins found to be altered significantly in both sexes for the mitochondrial dysfunction category were associated with Complex I (NADH:ubiquinone oxidoreductase) of the ETC; persistence of proteins in this complex was 76.90% in females and 26.34% in males.

**Table 1:** Average percent persistence per individual complex of the respiratory chain by sex

	Females			Males		
	%	*SD*	*n*	%	*SD*	*n*
CI	76.90	41.55	41	26.34	38.27	39
CII	67.61	22.01	3	19.00	49.78	4
CIII	94.25	78.93	9	−6.54	68.64	9
CIV	92.70	85.36	14	32.24	54.82	16
CV	145.75	108.29	8	143.24	238.64	7

Note

The four complexes of the ETC, along with ATP synthase, are shown along with the average percent persistence of all proteins found in each category. The standard deviation (*SD*) and the number of proteins in each complex (*n*) are also shown. The data were limited to eliminate outliers (Q1‐IQR*1.5 < protein percent persistence < Q3 + IQR*1.5).

One explanation for variation in the degree of persistence of proteins might be a relationship with protein half‐life, in that proteins with faster turnover might show less persistent changes after withdrawal of rapamycin treatment. However, we did not find any significant correlation between persistence of protein abundance changes due to rapamycin and our previously measured half‐lives of the same proteins in the heart (Dai et al., 2014; data not shown).

### 2.4 Rapamycin differentially alters respiratory chain complex activity by sex

Since rapamycin did persistently alter ETC Complex I (CI) peptide abundances for both sexes, we hypothesized that there may be a connection between ETC complex activity and diastolic function—that is, rapamycin might persistently alter the function of the ETC in both sexes and more specifically that rapamycin might change the ratio of complex activity to complex abundance, thereby changing the flux through the ETC. Electron flux is largely responsible for determining the mitochondrial membrane potential (ΔΨ_M_), by creating a proton gradient across in the inner mitochondrial membrane. An increase in ΔΨ_M_ has been correlated with an increase in the production of ROS in myocardium and in isolated cardiac mitochondria (Aon, Cortassa, & O'Rourke, 2008; Chen & Zweier, 2014). Rapamycin has previously been shown to lower ΔΨ_M_, through a mechanism independent of its well‐known cellular targets (Schieke et al., 2006). Thus, changing the ratio of complex activity to complex abundance might reduce the ΔΨ_M_ from a more pathological level in the aging heart, to a more physiological level. The impact of this could be reduced ROS, while maintaining appropriate ATP production, as needed for cardiac cycling activity. We therefore compared activity levels of the individual ETC complexes to the abundance of Complex proteins in the ETC, thereby obtaining a measure of activity/quantity of ETC protein.

**Figure 4:**
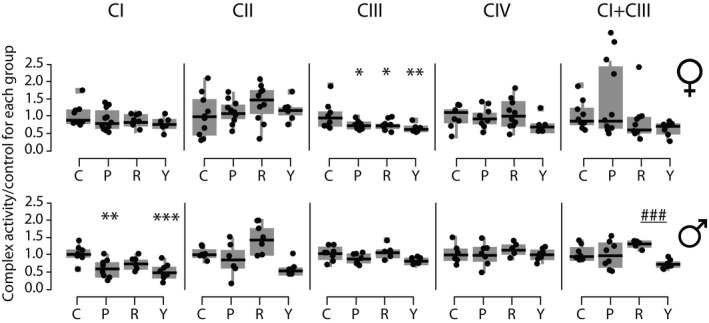
Rapamycin alters ETC Complex activity differentially by sex. (a) Boxplots of the activity in nmol/min mg^−1^ of each of the complexes, normalized to the activity of citrate synthase for each sample, then as a ratio against the control average activity per complex. Each data point is one mouse (average from technical triplicates). N per group is 8 to 16. C—control at 16 weeks, P—persistence, R—rapa, Y—young. Stars indicate significance, by Tukey's post hoc after an ANOVA of all groups per sex/complex which had an ANOVA *p* < .05, compared to control. *p < .05, **p < .01, ***p < .001

In the female cohorts, we were unable to detect significant differences in activity of CI, CII, and CIV between treatment groups (by ANOVA; Figure 4). There was, however, an age‐related increase in CIII activity in females, which was reduced persistently by rapamycin. Males showed a significant increase in CI activity with aging, which was partially reversed, again persistently, with rapamycin (Figure 4). Males also showed a significant increase in CII activity with rapamycin, though this was not persistent eight weeks after drug removal. All complex activity data were normalized to mitochondrial content, as determined by citrate synthase (CS) activity (see Figure S3). We also measured mitochondrial:nuclear DNA ratios in all samples and determined that mitochondrial content by this metric was similar between all old groups within each sex (Figure S3).

**Figure 5:**
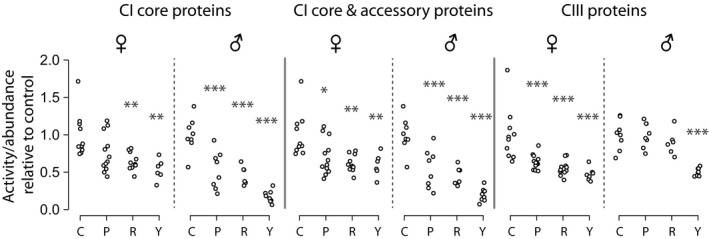
Rapamycin reduces ETC Complex I activity:Complex I protein ratios in both male and female mice, and Complex III activity:protein ratios in females. Calculation of activity to abundance ratio of protein was: (average individual complex activity per group divided by the average individual complex activity level of old control of same sex)/(average individual complex protein fold changes of each group divided by the old control). *p*‐values from one‐way ANOVAs performed per sex/complex, followed by Tukey's post hoc test when ANOVA was significant (after FDR correction). Each point is one animal. *p < .05, **p < .01, ***p < .001

While there was sexual dimorphism in the abundance and activity of components of ETC CI and CIII, both sexes consistently demonstrated a much greater ratio of activity:abundance in old controls than in young animals (Figure 5; see Discussion). The 16‐week rapamycin and the persistence treatment groups both showed a significant decline in the ratio of activity:abundance of CI, bringing levels toward that of the young animals. Inclusion of accessory components in addition to core components of CI did not substantially alter this effect. In CIII, females, but not males, showed a reversal of the age‐dependent increase in activity:abundance ratio in rapamycin and persistence groups (Figure 5). Consistent changes were not found in CII or CIV.

### 2.5 Metabolome differences seen at 8 weeks of rapamycin treatment are largely not persistent

Previous studies by our laboratory and others have shown evidence of an age‐related metabolic substrate switch from dependence on fatty acid oxidation (FAO) to glycolysis (Chiao et al., 2016; Wende, Brahma, McGinnis, & Young, 2017). Concordant with a more youthful function, we previously found evidence of a reversal of this aging substrate shift in mice treated for 10 weeks with rapamycin (Dai et al., 2014). This was confirmed by ^13^C glucose labeling and nuclear magnetic resonance in Langendorff‐perfused hearts (Chiao et al., 2016). In the present study, we found that the metabolomic changes after eight weeks of rapamycin treatment largely disappear after treatment with rapamycin is continued for a further eight weeks (Table 2). Categories shown in Table 2 were related to the metabolic substrate switch previously seen at 10 weeks of treatment with rapamycin. (See Figures S4 and S5 for more detailed information on the metabolite and enzyme abundances in the TCA cycle and glycolysis, respectively.) This does not appear to be simply a survivorship effect because the control hearts continue to show poor diastolic function at this later time point. It thus appears more likely that the rapamycin effects on cardiac metabolism are transient in nature. The metabolic shift may contribute to persistent cardiac remodeling, but it appears unlikely to be one of the major causes of the persistent functional changes that we observe (Figure 5).

**Table 2:**
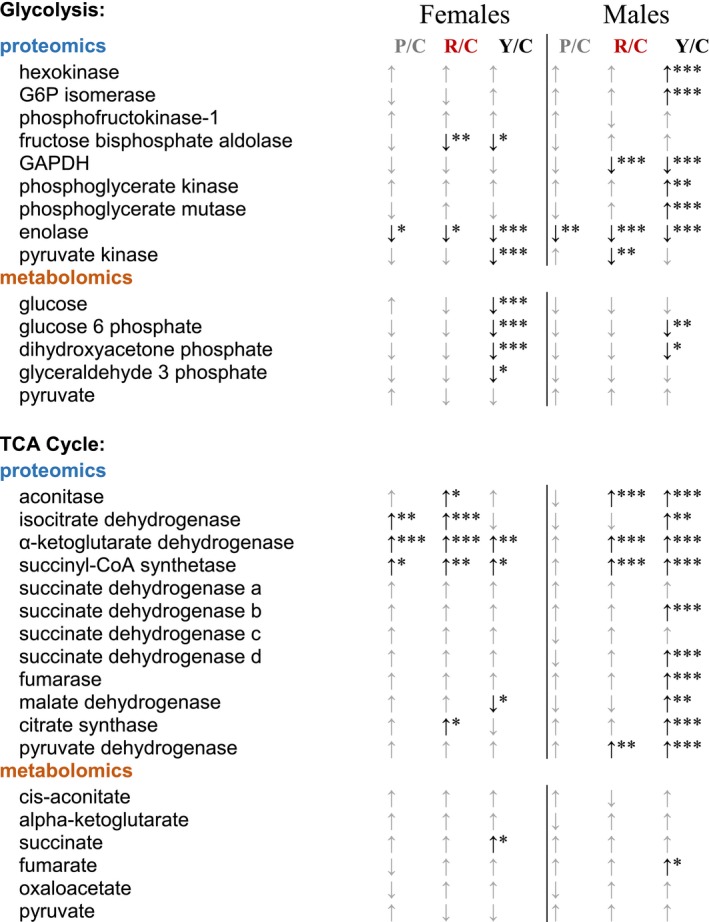
Metabolome changes due to aging are not persistently reversed by rapamycin

Note

P/C: persistence/control; R/C: rapa/control; Y/C: young/control. Up/down arrows indicate increased or decreased abundance compared to old control, respectively. Gray arrows—not significantly different by ANOVA. Significance was determined by one‐way ANOVA (metabolites) or two‐way ANOVA (proteins), and that with (FDR‐corrected) *p*‐values under .05 was further subjected to a Tukey post hoc test for group comparisons. *p < .05, **p < .01, ***p < .001.

## 3 DISCUSSION

Age‐related diastolic dysfunction is a pervasive problem with no current well‐targeted treatment options. Diastolic dysfunction limits cardiac reserve, which can lead to symptoms of exercise intolerance, and ultimately muscle wasting, loss of independence, and pulmonary venous congestion (Borlaug, 2014). Rapamycin has been shown to reverse age‐related diastolic dysfunction in rodents (Chiao et al., 2016; Luck, DeMarco, Mahmood, Gavini, & Pulakat, 2017) and dogs (Urfer et al., 2017). However, thus far there have not been studies that include both sexes and test for the persistence of the rapamycin‐dependent improvement. Our work indicates that in C57BL/6NIA mice, rapamycin can persistently improve diastolic function in aged animals of both sexes, even 8 weeks after cessation of an 8‐week treatment. We also explored possible mechanisms for this persistent benefit.

### 3.1 Echocardiography, hypertrophy, and stiffness

Both sexes of C57BL/6 mice show age‐related diastolic dysfunction, which is partially reversed by rapamycin treatment. This occurred in the absence of systolic changes in females and with only small changes in systolic function in males. Furthermore, this improvement was persistent for two months after drug removal. This is an exciting finding because it suggests that future treatments for diastolic dysfunction may be transient, potentially reducing cost, side effects (Pallet & Legendre, 2013; Verhave et al., 2014), and other negative effects of chronic drug treatment.

Rapamycin treatment also reduced cardiac hypertrophy, as previously found. Persistence of this effect was smaller than that of diastolic function, although it was significant when sexes were combined. Passive stiffness of the LV trabeculae was persistently reduced in the rapamycin‐treated animals. Reduction in hypertrophy and stiffness together indicate that at least some of the improvement in diastolic function was due to an improvement in passive filling (dependent on elastic properties of the tissue), rather than active (energy‐ and calcium sequestration‐dependent) relaxation of the left ventricle. This did not appear to be related to changes in cardiac fibrosis. Literature in the area of rapamycin and cardiac stiffness is sparse, but at least one paper suggested that rapamycin can decrease large artery stiffness in aged mice (Lesniewski et al., 2017). Further study is needed to determine which extracellular matrix or cellular changes are altered by rapamycin in the myocardium. Another potential source of increased passive stiffness is changes in myofilaments and their posttranslational modification, especially phosphorylation of key sites (Stienen, 2015); these also remain open for future study.

### 3.2 Proteomics and ETC activity

We interpreted the large proteomic abundance changes seen after 10‐week rapamycin treatment in previous experiments as evidence of improved proteostasis in the myocardium and postulated that these effects might remain persistent after cessation of the drug. Using shotgun proteomics (MS/MS), we found sex‐specific differences in protein abundance with rapamycin treatment, and while these differences were highly persistent in females after cessation of treatment, they were less so in males. The IPA pathway most significantly affected by rapamycin treatment in both sexes was mitochondrial dysfunction and oxidative phosphorylation. Within this category, many of the proteins found to be significantly altered were related to Complex I of the ETC, as either assembly factors, core proteins, or accessory proteins. This led us to hypothesize that there are activity‐level changes to the ETC, or at least to Complex I, with rapamycin treatment.

We found that both sexes had persistent decreases in ETC complex activities with rapamycin (Figure 4). CI activity was significantly reduced with rapamycin in male mice, and CIII was reduced in females. These changes were not coordinated with the abundance proteomics. However, we reasoned that the ratio of activity to abundance of proteins involved in CI and CIII might be coordinately altered by rapamycin treatment in both males and females, which proved to be the case (Figure 5). In both sexes, this change (a reduction in activity:abundance ratio) was also persistently reduced in CI, and a similar effect was seen in CIII in females. Thus, both the males and females may have accomplished a similar functional outcome *via* modulating the relationship of ETC activity and abundance.

Both CI and CIII have been implicated as prominent sources of ROS in mitochondria, and an increase in ΔΨ_M_ has also been shown to lead to an increase in ROS in myocardial cells, especially at higher ΔΨ_M_ (Zhou & Chen, 2014). This may be in part due to higher ΔΨ_M_ driving reverse electron flux through the respiratory chain, primarily through Complex I (Selivanov et al., 2011). Rapamycin has been previously demonstrated to reduce ΔΨ_M_ (Schieke et al., 2006). This information, especially combined with various clinical and animal model studies that demonstrate an increase in ROS generation in HF (Chen & Knowlton, 2010), suggests that in old hearts, rapamycin may persistently reduce the electron flux through the respiratory chain, leading to a reduced ROS generation, with subsequent beneficial effects.

There are additional reports that rapamycin may be abrogating levels of ROS through manipulation of CI and CIII. Martínez‐Cisuelo and colleagues published that 16‐month‐old mice showed age‐related increase in ROS production at CI in liver mitochondria, and this was abrogated by 7‐week rapamycin treatment (Martinez‐Cisuelo et al., 2016). Further, CIII ROS generation, at least in adipocyte differentiation, is dependent on mTORC1 signaling (Tormos et al., 2011). Still, more groups have found that mitochondrial ROS levels decrease with inhibition of TOR or mTORC1 (Xia, Sun, Xie, & Shu, 2017) and that this is associated with changes in ETC complex abundance (Pan & Shadel, 2009). Combined with previous work from our laboratory demonstrating a reduction in protein carbonylation in rapamycin‐treated old hearts (Dai et al., 2014), we speculate that the observed changes in CI and CIII activity and abundance may be coordinated to reduce mitochondrial ROS. Studies that directly test this hypothesis are needed to more fully understand the connection between ETC complex activity and abundance and rapamycin's organ‐wide functional improvement.

### 3.3 Metabolomics

Our laboratory has previously shown that 10‐week rapamycin treatment reverses the age‐related metabolic switch from dependence on FAO (beta‐oxidation, FAO) to glycolysis. Since this change was concurrent with improved diastolic function, we analyzed global metabolomics to see whether this shift was persistent 8 weeks after drug removal. While we found some evidence of the switch to glycolysis occurring in the cohorts in this study, those changes did not persist 8 weeks later—all old groups from both sexes (continuous rapamycin and persistence) were indistinguishable from each other at this time point. Thus, the metabolomes of the 16‐week rapamycin‐treated animals (28 month of age at that time) seemed to revert back to resembling the old control animals. Other studies have indicated that rapamycin's effects on glucose metabolism and insulin regulation are also phasic and reversible (Blum, 2002; Liu et al., 2014). While the metabolomic profile change that is evident within the first few weeks of rapamycin treatment (Chiao et al., 2016) may be important for diastolic functional improvement, it does not appear to be necessary for the persistent benefits. These data emphasize the importance at studying the kinetics of phenotypes, rather than single time points, when trying to determine possible mechanisms of drug effects.

## 4 CONCLUSION

We have found that rapamycin treatment in vivo leads to a persistent improvement in diastolic function, with mechanism that includes reduction of passive stiffness of the left ventricle, changes in the cardiac proteome, and a reduction in cardiac hypertrophy. Diastolic function can also be modulated by energy‐dependent processes, including calcium reuptake; we observed persistent changes in both sexes in the relationship of ETC activity and abundance, although how this may affect diastolic function needs to be more directly examined.

We also observed considerable sexual dimorphism in cellular and molecular changes due to rapamycin; thus, changes that are concordant and persistent in both sexes may provide a focus to best explain the mechanisms of rapamycin benefits, as well as those of other new therapies and more targeted approaches.

The goal of any treatment in humans is to maximize the benefits while minimizing the undesirable effects, with as low and infrequent of dosing as possible. To that end, many clinical studies have monitored the side effects of rapamycin treatment (Pallet & Legendre, 2013; Verhave et al., 2014) and animal model studies have sought to modulate those by either changing treatment duration or amount (Bitto et al., 2016; Miller et al., 2014). Most side effects of rapamycin are, however, reversible upon cessation of treatment (Kaplan, Qazi, & Wellen, 2014), although this was not addressed in the present study. Our work thus reinforces the approach of minimizing potential detrimental side effects of rapamycin treatment by utilizing an intermittent dosing regimen. Our results complement the prior body of work by showing that transient rapamycin treatment may be sufficient to confer a longer‐term health benefit.

## 5 EXPERIMENTAL PROCEDURES

### 5.1 Animals and husbandry

C57BL/6J female and male mice (3–28 months old) from the National Institute of Aging (NIA; originating from Charles River) were housed and maintained according to the guidelines of the Institutional Animal Care and Use Committee of the University of Washington. Both sexes began treatment at the 75% survival mark for the National Institutes of Aging colony of C57BL/6 mice (Turturro et al., 1999), 24 (male) or 22 (female) months of age. Animals were randomized and divided into three experimental groups. Treated animals received EUDRAGIT encapsulated rapamycin (purchased from the University of Texas Health Science Center, San Antonio) at 42 ppm (males) or 14 ppm (females) in standard chow (rapa group), or EUDRAGIT encapsulation alone in the chow (control group), for 8 or 16 weeks. The persistence group received rapamycin chow for 8 weeks, followed by control chow for a further 8 weeks. Young mice of the same genotype were acquired from the NIA at 3 months and used for the studies at 4 months of age. Animals were removed from the study whenever one of the several possible conditions was met: (a) loss of 20% body weight, (b) tumors or masses interfered with daily activity, or (c) inability to feed or drink with signs of impending death. Three of each old male group were censored. Censored old females included were six control, one rapa, and two persistence mice. No young animals were censored. Diet was prepared in house, by combining powdered standard rodent diet (LabDiet PicoLab Rodent Diet 20, #5053) with food coloring, agar, water, and either EUDRAGIT or encapsulated rapamycin. This mix was then compressed into patties and frozen until used. Animals had ad libitum access to food and water with a 12‐hr light/dark cycle. Experimental animals were euthanized by cervical dislocation. A 2‐mm mid‐vertical section was fixed in 4% formalin with the remainder of the tissue minced and flash‐frozen in liquid nitrogen (LN_2_). Frozen tissue was powdered with a TissueLyser II (Qiagen) and stored in LN_2_ until use.

### 5.2 Echocardiography

Longitudinal echocardiography occurred 0, 8, 12, and 16 weeks after start of treatment. Mice were anesthetized with 1%–2% isoflurane to keep the heart rate between 500 and 550 bpm. Breathing and heart rates were continuously monitored, and body temperature was kept stable using a circulating warm water pad. Images were captured using a 13 MHz probe with a Siemens Acuson CV‐70 (Siemens Medical Solution), using M‐mode and B‐mode views along with LV parasternal long‐axis view (D‐mode and TDI). Images taken when the heart rate was not within the 500–550 bpm range were excluded from analysis.

### 5.3 Proteomics

Flash‐frozen and pulverized heart tissues were processed and trypsin‐digested, and LC‐MS/MS analysis was performed with a Waters nanoAcquity UPLC and an Orbitrap Fusion Mass Spectrometer. For detailed methods on tissue processing, see Supplementary Methods.

Statistical analyses were performed using R (R Core Team, 2016) and Bioconductor (Fred Hutchinson Cancer Research Center). R scripts used in the proteomic analysis pipeline are available upon request from the corresponding author. Peptides that mapped to a single UniProt (Apweiler et al., 2004) protein accession for Mus musculus were used for quantification of protein abundance. A total of 31,791 entries (male dataset) and 27,165 entries (female dataset) from UniProtKB/*Swiss‐Prot* and UniProtKB/*TrEMBL* were found. Where one protein consisted of multiple peptides, statistical models were modified to appropriately account for this by using the peptides in each protein as a blocking factor. The total number of proteins found is as follows: 7,166 (males) and 5,870 (females). Statistically significant abundance changes in proteins between groups were determined by Student's *t* test, and *p*‐values were corrected for multiple testing by calculating *q*‐values; *q*‐values under 0.05 were considered statistically significant. See Supporting Information for detailed methods on pathway analysis and creation of heatmaps.

### 5.4 Passive stiffness

Experiments were performed as described in Moussavi‐Harami et al. (2015). In brief, excised hearts were demembranated overnight at 4°C in a relaxing solution (in mM: 100 KCl, 10 imidazole, 2 EGTA, 5 MgCl_2_, and 4 ATP) containing 50% glycerol (vol:vol), 1× protease inhibitor cocktail (Sigma‐Aldrich, P8340), and 1% Triton X‐100. Hearts were then stored at −20°C in the same solution without Triton X‐100 for up to 1 week. LV multicellular tissues were dissected out and wrapped between aluminum t‐clips, then mounted between a motor (Aurora Scientific, Model 312B) and force transducer (Aurora Scientific, Model 403A). Preparations were moved to a bath containing experimental relaxing solution at pH 7.0 and 15°C containing (in mM): 15 phosphocreatine, 15 EGTA, 80 MOPS, 1 free Mg^2+^, 10^–6^ Ca^2+^, 1 DTT, and 5 Mg_2_ ATP. Preparations were set to length just above slack (L_0_), then sequentially stretched at 4% increments up to 24% L_0_. We calculated median passive force (mN/mm^2^) per mouse from multiple preparations (1–4 per heart). We scaled all data to controls at 24% length change for each experiment to compare 3 experiments at once. *N* = 3–12 mice per group. Scaled data were log‐transformed, and linear modeling was performed. *p*‐values are from linear modeling. All groups have both sexes combined. We did not observe sex‐related differences.

### 5.5 Picrosirius red staining

Formalin‐fixed/paraffin‐embedded mid‐ventricle sections from each heart were counterstained to visualize nuclei with Weigert's hematoxylin (MilliporeSigma) and then stained with 0.1% sirius red in saturated picric acid (Rowley Biochemical). Slides were briefly washed in acidified water (87 mmol glacial acetic acid), dehydrated, and mounted in Permount (Fisher Scientific). All images were obtained with the same exposure time, white balance, and saturation on a Leica DMLS outfitted with a circular polarizer and a AmScope MU 300 camera.

Positive picrosirius red signal and total tissue area were quantified in ImageJ. Percent positive signal was determined for each image (*n* = 8–22 images per heart), and median % positive signal was calculated per animal. N per group: C‐6, P‐6, R‐4, and Y‐2.

### 5.6 Metabolite extraction and analysis

Metabolic profiling by LC‐MS was performed as previously described (Dai et al., 2014). For detailed tissue extraction methods, see Supplementary Methods.

Data were log2‐transformed, centered and scaled (by standard deviation), and then analyzed using one‐way ANOVA per sex and metabolite in R. Sample sizes for each group (old control, old rapa, old persistence, and young controls) were 8, 8, 8, and 6 (females) and 8, 7, 8, and 7 (males).

### 5.7 Statistical analysis

For specific statistical analyses, see each methods subsection. In general, all tests involving one sex with more than two groups were one‐way ANOVAs. Two‐way ANOVA with repeated measures was used, when one sex with more than two groups was tested over multiple time points (i.e., for Ea/Aa ratios). Following ANOVAs, Tukey's post hoc tests were used to determine group–group differences. Student's *t* tests or Welch two‐sample tests were used when comparing two groups. Multiple testing corrections were performed using the Bioconductor package *q*‐value. *p*‐values and *q*‐values less than .05 were considered statistically significant.

## CONFLICT OF INTEREST

The authors have no conflicts of interest to declare.

## AUTHORS' CONTRIBUTIONS

EQ and PSR designed the study. EQ and JF performed research. MTS, NN, and JF contributed to histological work. MR, KK, FMH, and MR performed biomechanics. GM and HG performed proteomic and metabolomic mass spectrometry. EQ, CQ, and NB provided statistical and R code expertise. YAC provided training and quality control for echocardiography.

## Data Availability

The data that support the findings of this study are openly available in GitHub at https://github.com/EllenQuarles/RapamycinPersistence.

## References

[acel13086-bib-0001] Aon, M. A. , Cortassa, S. , & O'Rourke, B. (2008). Mitochondrial oscillations in physiology and pathophysiology. Advances in Experimental Medicine and Biology, 641, 98–117. 18783175 10.1007/978-0-387-09794-7_8 PMC2692514

[acel13086-bib-0002] Apweiler, R. , Bairoch, A. , Wu, C. H. , Barker, W. C. , Boeckmann, B. , Ferro, S. , … Yeh, L. S. (2004). UniProt: The Universal Protein knowledgebase. Nucleic Acids Research, 32, D115–D119. 10.1093/nar/gkh131 14681372 PMC308865

[acel13086-bib-0003] Asif, M. , Egan, J. , Vasan, S. , Jyothirmayi, G. N. , Masurekar, M. R. , Lopez, S. , … Regan, T. J. (2000). An advanced glycation endproduct cross‐link breaker can reverse age‐related increases in myocardial stiffness. Proceedings of the National Academy of Sciences of the United States of America, 97, 2809–2813. 10.1073/pnas.040558497 10706607 PMC16011

[acel13086-bib-0004] Bitto, A. , Ito, T. K. , Pineda, V. V. , LeTexier, N. J. , Huang, H. Z. , Sutlief, E. , … Kaeberlein, M. (2016). Transient rapamycin treatment can increase lifespan and healthspan in middle‐aged mice. eLife, 5:e16351 10.7554/eLife.16351 27549339 PMC4996648

[acel13086-bib-0005] Bitto, A. , Wang, A. M. , Bennett, C. F. , & Kaeberlein, M. (2015). Biochemical genetic pathways that modulate aging in multiple species. Cold Spring Harbor Perspectives in Medicine, 5. pii: a025114. 10.1101/cshperspect.a025114 PMC4632857 26525455

[acel13086-bib-0006] Blum, C. B. (2002). Effects of sirolimus on lipids in renal allograft recipients: An analysis using the Framingham risk model. American Journal of Transplantation, 2, 551–559. 10.1034/j.1600-6143.2002.20610.x 12118900

[acel13086-bib-0007] Boers‐Doets, C. B. , Raber‐Durlacher, J. E. , Treister, N. S. , Epstein, J. B. , Arends, A. B. , Wiersma, D. R. , … Gelderblom, H. (2013). Mammalian target of rapamycin inhibitor‐associated stomatitis. Future Oncology, 9, 1883–1892. 24295418 10.2217/fon.13.141

[acel13086-bib-0008] Borlaug, B. A. (2014). The pathophysiology of heart failure with preserved ejection fraction. Nature Reviews Cardiology, 11, 507–515. 10.1038/nrcardio.2014.83 24958077

[acel13086-bib-0009] Campbell, K. S. , & Sorrell, V. L. (2015). Cell‐ and molecular‐level mechanisms contributing to diastolic dysfunction in HFpEF. Journal of Applied Physiology, 119, 1228–1232. 10.1152/japplphysiol.01168.2014 25911687 PMC4816411

[acel13086-bib-0010] Chang, P. P. , Chambless, L. E. , Shahar, E. , Bertoni, A. G. , Russell, S. D. , Ni, H. , … Rosamond, W. D. (2014). Incidence and survival of hospitalized acute decompensated heart failure in four US communities (from the Atherosclerosis Risk in Communities Study). American Journal of Cardiology, 113, 504–510. 10.1016/j.amjcard.2013.10.032 24342763 PMC4038413

[acel13086-bib-0011] Chen, L. , & Knowlton, A. A. (2010). Mitochondria and heart failure: New insights into an energetic problem. Minerva Cardioangiologica, 58, 213–229. 20440251 PMC3786553

[acel13086-bib-0012] Chen, Y. R. , & Zweier, J. L. (2014). Cardiac mitochondria and reactive oxygen species generation. Circulation Research, 114, 524–537. 10.1161/CIRCRESAHA.114.300559 24481843 PMC4118662

[acel13086-bib-0013] Chiao, Y. A. , Kolwicz, S. C. , Basisty, N. , Gagnidze, A. , Zhang, J. , Gu, H. , … Rabinovitch, P. S. (2016). Rapamycin transiently induces mitochondrial remodeling to reprogram energy metabolism in old hearts. Aging, 8, 314–327. 10.18632/aging.100881 26872208 PMC4789585

[acel13086-bib-0014] Chiao, Y. A. , & Rabinovitch, P. S. (2015). The aging heart. Cold Spring Harbor Perspectives in Medicine, 5, a025148. 26328932 10.1101/cshperspect.a025148 PMC4561390

[acel13086-bib-0015] Dai, D. F. , Karunadharma, P. P. , Chiao, Y. A. , Basisty, N. , Crispin, D. , Hsieh, E. J. , … Rabinovitch, P. S. (2014). Altered proteome turnover and remodeling by short‐term caloric restriction or rapamycin rejuvenate the aging heart. Aging Cell, 13, 529–539. 10.1111/acel.12203 24612461 PMC4040127

[acel13086-bib-0016] Dai, D. F. , & Rabinovitch, P. S. (2009). Cardiac aging in mice and humans: The role of mitochondrial oxidative stress. Trends in Cardiovascular Medicine, 19, 213–220. 10.1016/j.tcm.2009.12.004. 20382344 PMC2858758

[acel13086-bib-0017] Das, A. , Durrant, D. , Koka, S. , Salloum, F. N. , Xi, L. , & Kukreja, R. C. (2014). Mammalian target of rapamycin (mTOR) inhibition with rapamycin improves cardiac function in type 2 diabetic mice: Potential role of attenuated oxidative stress and altered contractile protein expression. Journal of Biological Chemistry, 289, 4145–4160. 10.1074/jbc.M113.521062 24371138 PMC3924280

[acel13086-bib-0018] Fine, N. M. , & Kushwaha, S. S. (2016). Recent advances in mammalian target of rapamycin inhibitor use in heart and lung transplantation. Transplantation, 100, 2558–2568. 10.1097/TP.0000000000001432 27495747

[acel13086-bib-0019] Flynn, J. M. , O'Leary, M. N. , Zambataro, C. A. , Academia, E. C. , Presley, M. P. , Garrett, B. J. , … Melov, S. (2013). Late‐life rapamycin treatment reverses age‐related heart dysfunction. Aging Cell, 12, 851–862. 10.1111/acel.12109 23734717 PMC4098908

[acel13086-bib-0020] Harrison, D. E. , Strong, R. , Sharp, Z. D. , Nelson, J. F. , Astle, C. M. , Flurkey, K. , … Miller, R. A. (2009). Rapamycin fed late in life extends lifespan in genetically heterogeneous mice. Nature, 460, 392–395. 10.1038/nature08221 19587680 PMC2786175

[acel13086-bib-0021] Heidenreich, P. A. , Albert, N. M. , Allen, L. A. , Bluemke, D. A. , Butler, J. , Fonarow, G. C. , … Trogdon, J. G. (2013). Forecasting the impact of heart failure in the United States: A policy statement from the American Heart Association. Circulation: Heart Failure, 6, 606–619. 10.1161/HHF.0b013e318291329a 23616602 PMC3908895

[acel13086-bib-0022] Jalil, J. E. , Doering, C. W. , Janicki, J. S. , Pick, R. , Shroff, S. G. , & Weber, K. T. (1989). Fibrillar collagen and myocardial stiffness in the intact hypertrophied rat left ventricle. Circulation Research, 64, 1041–1050. 2524288 10.1161/01.res.64.6.1041

[acel13086-bib-0023] Kaplan, B. , Qazi, Y. , & Wellen, J. R. (2014). Strategies for the management of adverse events associated with mTOR inhibitors. Transplantation Reviews, 28, 126–133. 24685370 10.1016/j.trre.2014.03.002

[acel13086-bib-0024] Lesniewski, L. A. , Seals, D. R. , Walker, A. E. , Henson, G. D. , Blimline, M. W. , Trott, D. W. , … Donato, A. J. (2017). Dietary rapamycin supplementation reverses age‐related vascular dysfunction and oxidative stress, while modulating nutrient‐sensing, cell cycle, and senescence pathways. Aging Cell, 16, 17–26. 10.1111/acel.12524 27660040 PMC5242306

[acel13086-bib-0025] Liu, Y. , Diaz, V. , Fernandez, E. , Strong, R. , Ye, L. , Baur, J. A. , … Salmon, A. B. (2014). Rapamycin‐induced metabolic defects are reversible in both lean and obese mice. Aging, 6, 742–754. 10.18632/aging.100688 25324470 PMC4221917

[acel13086-bib-0026] Luck, C. , DeMarco, V. G. , Mahmood, A. , Gavini, M. P. , & Pulakat, L. (2017). Differential regulation of cardiac function and intracardiac cytokines by rapamycin in healthy and diabetic rats. Oxidative Medicine and Cellular Longevity, 2017, 5724046 10.1155/2017/5724046 28408970 PMC5376943

[acel13086-bib-0027] Martinez‐Cisuelo, V. , Gomez, J. , Garcia‐Junceda, I. , Naudi, A. , Cabre, R. , Mota‐Martorell, N. , … Barja, G. (2016). Rapamycin reverses age‐related increases in mitochondrial ROS production at complex I, oxidative stress, accumulation of mtDNA fragments inside nuclear DNA, and lipofuscin level, and increases autophagy, in the liver of middle‐aged mice. Experimental Gerontology, 83, 130–138. 10.1016/j.exger.2016.08.002 27498120

[acel13086-bib-0028] Miller, R. A. , Harrison, D. E. , Astle, C. M. , Baur, J. A. , Boyd, A. R. , de Cabo, R. , … Strong, R. (2011). Rapamycin, but not resveratrol or simvastatin, extends life span of genetically heterogeneous mice. Journals of Gerontology. Series A, Biological Sciences and Medical Sciences, 66, 191–201. 10.1093/gerona/glq178 PMC3021372 20974732

[acel13086-bib-0029] Miller, R. A. , Harrison, D. E. , Astle, C. M. , Fernandez, E. , Flurkey, K. , Han, M. , … Strong, R. (2014). Rapamycin‐mediated lifespan increase in mice is dose and sex dependent and metabolically distinct from dietary restriction. Aging Cell, 13, 468–477. 10.1111/acel.12194 24341993 PMC4032600

[acel13086-bib-0030] Moran, A. E. , Forouzanfar, M. H. , Roth, G. A. , Mensah, G. A. , Ezzati, M. , Flaxman, A. , … Naghavi, M. (2014). The global burden of ischemic heart disease in 1990 and 2010: The Global Burden of Disease 2010 study. Circulation, 129, 1493–1501. 10.1161/CIRCULATIONAHA.113.004046 24573351 PMC4181601

[acel13086-bib-0031] Moussavi‐Harami, F. , Razumova, M. V. , Racca, A. W. , Cheng, Y. , Stempien‐Otero, A. , & Regnier, M. (2015). 2‐Deoxy adenosine triphosphate improves contraction in human end‐stage heart failure. Journal of Molecular and Cellular Cardiology, 79, 256–263. 25498214 10.1016/j.yjmcc.2014.12.002 PMC4301986

[acel13086-bib-0032] Pallet, N. , & Legendre, C. (2013). Adverse events associated with mTOR inhibitors. Expert Opinion on Drug Safety, 12, 177–186. 10.1517/14740338.2013.752814 23252795

[acel13086-bib-0033] Pan, Y. , & Shadel, G. S. (2009). Extension of chronological life span by reduced TOR signaling requires down‐regulation of Sch9p and involves increased mitochondrial OXPHOS complex density. Aging, 1, 131–145. 10.18632/aging.100016 20157595 PMC2815770

[acel13086-bib-0034] Park, K. W. , Kang, S. H. , Velders, M. A. , Shin, D. H. , Hahn, S. , Lim, W. H. , … Kim, H. S. (2013). Safety and efficacy of everolimus‐ versus sirolimus‐eluting stents: A systematic review and meta‐analysis of 11 randomized trials. American Heart Journal, 165, 241–50.e4. 10.1016/j.ahj.2012.08.007 23351828

[acel13086-bib-0035] Paul, D. S. , Grevengoed, T. J. , Pascual, F. , Ellis, J. M. , Willis, M. S. , & Coleman, R. A. (2014). Deficiency of cardiac Acyl‐CoA synthetase‐1 induces diastolic dysfunction, but pathologic hypertrophy is reversed by rapamycin. Biochimica Et Biophysica Acta, 1841, 880–887. 10.1016/j.bbalip.2014.03.001 24631848 PMC4047709

[acel13086-bib-0036] Plitt, G. D. , Spring, J. T. , Moulton, M. J. , & Agrawal, D. K. (2018). Mechanisms, diagnosis, and treatment of heart failure with preserved ejection fraction and diastolic dysfunction. Expert Review of Cardiovascular Therapy, 16, 579–589. 29976104 10.1080/14779072.2018.1497485 PMC6287909

[acel13086-bib-0038] R Core Team . (2016). R: A language and environment for statistical computing. Available at: https://www.r-project.org/.

[acel13086-bib-0039] Schieke, S. M. , Phillips, D. , McCoy, J. P. Jr. , Aponte, A. M. , Shen, R. F. , Balaban, R. S. , & Finkel, T. (2006). The mammalian target of rapamycin (mTOR) pathway regulates mitochondrial oxygen consumption and oxidative capacity. Journal of Biological Chemistry, 281, 27643–27652. 10.1074/jbc.M603536200 16847060

[acel13086-bib-0040] Selivanov, V. A. , Votyakova, T. V. , Pivtoraiko, V. N. , Zeak, J. , Sukhomlin, T. , Trucco, M. , … Cascante, M. (2011). Reactive oxygen species production by forward and reverse electron fluxes in the mitochondrial respiratory chain. PLoS Computational Biology, 7, e1001115 10.1371/journal.pcbi.1001115 21483483 PMC3068929

[acel13086-bib-0041] Stienen, G. J. M. (2015). Pathomechanisms in heart failure: The contractile connection. Journal of Muscle Research and Cell Motility, 36, 47–60. 10.1007/s10974-014-9395-8 25376563

[acel13086-bib-0042] Tormos, K. V. , Anso, E. , Hamanaka, R. B. , Eisenbart, J. , Joseph, J. , Kalyanaraman, B. , & Chandel, N. S. (2011). Mitochondrial complex III ROS regulate adipocyte differentiation. Cell Metabolism, 14, 537–544. 10.1016/j.cmet.2011.08.007 21982713 PMC3190168

[acel13086-bib-0043] Turturro, A. , Witt, W. W. , Lewis, S. , Hass, B. S. , Lipman, R. D. , & Hart, R. W. (1999). Growth curves and survival characteristics of the animals used in the Biomarkers of Aging Program. Journals of Gerontology. Series A, Biological Sciences and Medical Sciences, 54, B492–B501. 10.1093/gerona/54.11.B492 10619312

[acel13086-bib-0044] Urfer, S. R. , Kaeberlein, T. L. , Mailheau, S. , Bergman, P. J. , Creevy, K. E. , Promislow, D. E. L. , & Kaeberlein, M. (2017). A randomized controlled trial to establish effects of short‐term rapamycin treatment in 24 middle‐aged companion dogs. Geroscience, 39, 117–127. 10.1007/s11357-017-9972-z 28374166 PMC5411365

[acel13086-bib-0045] Verhave, J. , Boucher, A. , Dandavino, R. , Collette, S. , Senecal, L. , Hebert, M. J. , … Cardinal, H. (2014). The incidence, management, and evolution of rapamycin‐related side effects in kidney transplant recipients. Clinical Transplants, 28, 616–622. 10.1111/ctr.12361 24654608

[acel13086-bib-0046] Wende, A. R. , Brahma, M. K. , McGinnis, G. R. , & Young, M. E. (2017). Metabolic origins of heart failure. JACC: Basic to Translational Science, 2, 297–310. 10.1016/j.jacbts.2016.11.009 28944310 PMC5609457

[acel13086-bib-0047] Xia, Y. , Sun, M. , Xie, Y. , & Shu, R. (2017). mTOR inhibition rejuvenates the aging gingival fibroblasts through alleviating oxidative stress. Oxidative Medicine and Cellular Longevity, 2017, 6292630. 28804534 10.1155/2017/6292630 PMC5540269

[acel13086-bib-0048] Zhang, Y. , Bokov, A. , Gelfond, J. , Soto, V. , Ikeno, Y. , Hubbard, G. , … Fischer, K. (2014). Rapamycin extends life and health in C57BL/6 mice. Journals of Gerontology. Series A, Biological Sciences and Medical Sciences, 69, 119–130. 10.1093/gerona/glt056 PMC4038246 23682161

[acel13086-bib-0049] Zhou, X. , & Chen, J. (2014). Is treatment with trimetazidine beneficial in patients with chronic heart failure? PLoS ONE, 9, e94660 10.1371/journal.pone.0094660 24797235 PMC4010408

