# Supplementary material for: Rapamycin persistently improves cardiac function in aged, male and female mice, even following cessation of treatment
Source: Aging Cell. 2019 Dec 10;19(2):e13086. doi: 10.1111/acel.13086 (PMC6996961; doi:10.1111/acel.13086)
Supplement: Supplementary file 1 [file ACEL-19-e13086-s001.docx]

**11. Supporting Information**

**Supporting Methods**

**Pathway Analysis and Heatmaps**

We used Ingenuity Pathway Analysis (IPA®, QIAGEN Redwood City, www.qiagen.com/ingenuity) to generate lists of pathways that were significantly affected by rapamycin. Multiple testing corrections, to control for estimated false discovery rate, were performed using the Bioconductor q-value package. Only proteins whose expression was significantly different by Student’s t-test, (threshold of q-value < 0.05), between Control and Rapamycin (continuous) groups were included in the queries to identify canonical pathways from IPA. IPA uses a Fisher’s exact test to determine the p-value of enrichment into canonical pathways, after deriving the significance of the association between the data set and a pathway (number molecules from dataset fitting into a pathway/total number of molecules present in curated pathway). Z-scores of abundance of proteins in each pathway, and all significantly affected proteins per sex, were visualized on heatmaps created in R using the heatmaps.2 function in the gplots (Warnes et al. 2016) package. Clustering in Dendrograms was performed using Ward’s method.

**Tissue Processing for Proteomics**

Flash-frozen and pulverized heart tissues were sonicated in ice-cold 50 mM ammonium bicarbonate + 0.1% (wt/vol) *Rapi*Gest (Waters Corporation, product number 186001860), then centrifuged at 8000 g for 10 min at 4C. Concentration of soluble protein in the supernatant was determined by BCA (ThermoFisher Scientific, cat # 23225). DTT was added to 20 μg of protein to a final concentration of 5 mM, and incubated at 50°C for 30 minutes. After cooling to room temperature, iodoacetamide (IAA) wsa added to a final concentration of 15 mM and incubated in the dark for 30 minutes. Trypsin (sequencing grade, Promega, cat #), was added at a ratio of 1:50 enzyme:protein, incubated 2 hrs in dark while shaking at 37°C. Typsin was neutralized with HCl at 200 mM, incubated with shaking for 45 minutes at 37°C. Samples were centrifuged at 16k g, at 4°C, for 20 minutes. Supernatant was cleaned with preconditioned MCX columns (company, cat #), washing with 0.1% formic acid in water, 90% acetonitrile:10% water. Peptides were eluted with 600 μL 2.8% NH4OH in methanol. Peptides were dried under vacuum until only a few μL were left per sample, after which they were reconstituted with 160 μL 0.1% formic acid in water and stored at 4°C until LC‐MS/MS analysis was performed with a Waters nanoAcquity UPLC and an Orbitrap Fusion Mass Spectrometer.

**Metabolite Extraction**

10-12 mg of flash-frozen and pulverized heart tissue was homogenized in 200 μL water:HPLC grade methanol (1:4, -75°C, on dry ice). To each sample, we added 800 μL water:methanol (1:4) and incubated for 30 minutes on dry ice. Then the samples were centrifuged at 14K rpm for 5 minutes at 4°C. Supernatants were saved on dry ice. Pellets were resuspended in 500 μL water:HPLC grade methanol (1:4) and incubated for 15 minutes on dry ice. These were centrifuged again, 14k rpm, 5 minutes, at 4°C and the soluble extract was combined with the first supernatant. Samples were dried completely under vacuum at 30°C.

**References in Supporting Methods**

Warnes G, Bolker B, Bonebakker L, Gentleman R, Huber W, Liaw A, Lumley T, Maechler M, Magnusson, Arni, Moeller S, Schwartz M & Venables B (2016) gplots: Various R Programming Tools for Plotting Data . R package version 3.0.1. Available at: https://cran.r-project.org/package=gplots.

***

*

**

**Supporting Figure 1. Heart mass:tibia length is significantly persistent in data combining both sexes.** The heart mass:tibia length data from Figure 1C were normalized to the old control groups for each sex, then combined and used for T-tests. *p<0.05, **p<0.01, ***p<0.001. N=15~22 per group.

Supporting Table 1. Gene names from female heatmap in Figure 2A.

|  | z-scores | | |
| --- | --- | --- | --- |
|  | Control | Rapamycin | Persistence |
| Atp5f1 | -1.0407 | 0.0870 | 0.9537 |
| Ndufs2 | -1.1314 | 0.3657 | 0.7657 |
| Vdac1 | -1.1494 | 0.4785 | 0.6708 |
| Ndufb4 | -1.1507 | 0.4918 | 0.6589 |
| Ndufs1 | -1.1543 | 0.5504 | 0.6039 |
| Ndufb8 | -1.1542 | 0.5480 | 0.6062 |
| Ogdh | -1.1531 | 0.5244 | 0.6288 |
| Ndufa2 | -1.1535 | 0.5314 | 0.6221 |
| Atp5o | -0.8672 | 1.0939 | -0.2266 |
| Cpt1b | -1.0016 | 0.9984 | 0.0032 |
| Sdhd | -1.0798 | 0.8942 | 0.1855 |
| Uqcrc1 | -1.0444 | 0.9487 | 0.0956 |
| Ndufb11 | -1.1484 | 0.6786 | 0.4698 |
| Cox7a1 | -1.1448 | 0.7030 | 0.4419 |
| Cox6a2 | -1.1470 | 0.6888 | 0.4582 |
| Vdac2 | -1.1463 | 0.6938 | 0.4525 |
| Vdac3 | -1.1014 | 0.8510 | 0.2504 |
| Cyc1 | -1.1115 | 0.8268 | 0.2847 |
| Ndufa9 | -1.1097 | 0.8313 | 0.2785 |
| Ndufa4 | -1.1360 | 0.7473 | 0.3887 |
| Cox6c | -1.1397 | 0.7303 | 0.4094 |
| Ndufa3 | -1.1234 | 0.7928 | 0.3306 |
| Ndufb7 | -1.1330 | 0.7596 | 0.3733 |
| Ndufa10 | -1.1313 | 0.7662 | 0.3651 |
| Ndufa8 | -1.1302 | 0.7699 | 0.3604 |
| Cox5a | 0.4012 | 0.7371 | -1.1383 |
| Ndufab1 | -0.1331 | 1.0599 | -0.9268 |
| Txn2 | 1.0652 | -0.9186 | -0.1466 |
| Ndufv2 | 1.1270 | -0.7812 | -0.3459 |
| Atp5j | 0.9127 | 0.1562 | -1.0689 |
| Ndufs6 | 1.1084 | -0.2739 | -0.8345 |
| Ndufv3 | 1.0919 | -0.2205 | -0.8713 |
| Prdx3 | 1.1439 | -0.4354 | -0.7085 |
| Atp5d | 1.1538 | -0.5376 | -0.6163 |
| Myl9 | 0.9775 | 0.0435 | -1.0210 |
| Myl1 | 1.0293 | -0.0614 | -0.9679 |
| Myl7 | 1.0222 | -0.0461 | -0.9762 |
| Myl3 | 1.0877 | -0.2082 | -0.8795 |
| Ppp1r7 | 1.0918 | -0.2204 | -0.8714 |
| Myl4 | 1.1248 | -0.3364 | -0.7885 |
| Myl2 | 1.1083 | -0.2735 | -0.8348 |
| Ywhag | 1.0476 | -0.9444 | -0.1033 |
| Akap12 | 1.0831 | -0.8881 | -0.1950 |
| Tnni3 | 1.1547 | -0.5815 | -0.5732 |
| Ywhah | 1.1517 | -0.6482 | -0.5035 |
| Ywhaz | 1.1422 | -0.4244 | -0.7178 |
| Pgp | 1.1423 | -0.4253 | -0.7171 |
| Ywhae | 1.1471 | -0.4592 | -0.6879 |
| Ppp1cb | 1.1500 | -0.4850 | -0.6650 |
| Myl6 | 1.1519 | -0.5060 | -0.6459 |
| Ctnnb1 | -0.1722 | 1.0749 | -0.9027 |
| Pygb | -0.8365 | 1.1076 | -0.2712 |
| Camk2d | -0.5567 | -0.5978 | 1.1545 |
| Ttn | -0.8230 | -0.2899 | 1.1129 |
| Ryr2 | -1.1089 | 0.2757 | 0.8333 |
| Gnb2 | -1.1543 | 0.6027 | 0.5516 |
| Flnc | -1.1545 | 0.5967 | 0.5578 |
| Naca | 1.0164 | -0.0336 | -0.9828 |
| Myh9 | 1.0240 | -0.0500 | -0.9741 |
| Cfl1 | 1.1335 | -0.3759 | -0.7576 |
| Vim | 1.1420 | -0.7190 | -0.4230 |
| Actn2 | -1.1318 | 0.7642 | 0.3676 |
| Ppp2r1a | -1.1420 | 0.4230 | 0.7190 |
| Myh13 | 0.6694 | -1.1495 | 0.4802 |
| Acta1 | 0.9987 | -1.0013 | 0.0027 |
| Dsp | -0.8631 | -0.2327 | 1.0958 |
| Myh6 | -0.4582 | -0.6888 | 1.1470 |
| Myh7 | -0.1378 | -0.9240 | 1.0617 |
| Calr | 0.8914 | 0.1900 | -1.0814 |
| Tpm4 | 1.1546 | -0.5668 | -0.5878 |
| Tpm1 | 1.1494 | -0.4790 | -0.6705 |
| Tnnt2 | 1.1311 | -0.3645 | -0.7666 |
| Atp2a2 | -1.1519 | 0.5067 | 0.6452 |
| Kng1 | 0.8522 | 0.2487 | -1.1009 |
| Vav1 | -1.1546 | 0.5619 | 0.5927 |
| Rac1 | -1.1417 | 0.4210 | 0.7207 |
| Clip1 | 0.2749 | 0.8338 | -1.1087 |
| Jup | -1.0836 | 0.1964 | 0.8872 |
| Sorbs1 | -1.1301 | 0.3596 | 0.7704 |
| Map3k10 | 1.0472 | -0.1023 | -0.9449 |
| Cdh13 | 1.1047 | -0.2612 | -0.8435 |
| Arhgef17 | 1.1376 | -0.3973 | -0.7403 |
| Sptan1 | 1.1384 | -0.4017 | -0.7367 |
| Arhgdib | 0.9707 | -1.0269 | 0.0562 |
| Arhgdia | 0.7555 | 0.3785 | -1.1340 |
| Nppa | 0.7671 | 0.3639 | -1.1310 |

Supporting Table 2. Gene names from male heatmap in Figure 2A.

|  | z-scores | | |
| --- | --- | --- | --- |
|  | Control | Rapamycin | Persistence |
| Atp5c1 | -0.0888 | -0.9526 | 1.0414 |
| Ndufab1 | 0.5678 | -1.1546 | 0.5869 |
| Ndufs4 | 0.2629 | -1.1052 | 0.8423 |
| Snca | 0.8572 | -1.0986 | 0.2415 |
| Prdx5 | 0.7841 | -1.1261 | 0.3420 |
| Sod2 | 0.9887 | -1.0109 | 0.0222 |
| Ndufs6 | 0.9958 | -1.0041 | 0.0083 |
| Atp5j | 0.9633 | -1.0330 | 0.0697 |
| Atp5b | 0.9307 | -1.0573 | 0.1266 |
| Txn2 | 0.9433 | -1.0484 | 0.1051 |
| Cox5a | 1.1408 | -0.7253 | -0.4154 |
| Ndufv3 | 1.1499 | -0.6658 | -0.4841 |
| Prdx3 | 1.0558 | -0.9328 | -0.1229 |
| Ndufv2 | 1.0650 | -0.9189 | -0.1462 |
| Atp5d | 1.1177 | -0.8100 | -0.3077 |
| Atp5e | 1.1044 | -0.8440 | -0.2604 |
| Xdh | 1.1083 | -0.8348 | -0.2735 |
| Cyc1 | -0.7916 | 1.1238 | -0.3322 |
| Uqcrc1 | -0.8158 | 1.1156 | -0.2998 |
| Ndufa9 | -0.8243 | 1.1124 | -0.2881 |
| Ndufa10 | -0.8638 | 1.0955 | -0.2317 |
| Cox7a2l | -0.8482 | 1.1026 | -0.2545 |
| Park7 | -0.8534 | 1.1003 | -0.2469 |
| Ndufb5 | -0.8893 | 1.0825 | -0.1932 |
| Ndufa8 | -0.9054 | 1.0733 | -0.1680 |
| Mt-Cyb | -1.0348 | 0.9612 | 0.0736 |
| Ndufb8 | -1.0347 | 0.9613 | 0.0734 |
| Ndufa1 | -1.0931 | 0.8688 | 0.2243 |
| Cox7b | -1.0605 | 0.9258 | 0.1347 |
| Sdha | -1.0624 | 0.9230 | 0.1393 |
| Ndufb6 | -0.9358 | 1.0537 | -0.1179 |
| Ndufa12 | -0.9429 | 1.0487 | -0.1058 |
| Fis1 | -0.9456 | 1.0467 | -0.1012 |
| Maob | -1.0114 | 0.9882 | 0.0231 |
| Ndufb7 | -0.9951 | 1.0048 | -0.0098 |
| Ogdh | -0.9777 | 1.0209 | -0.0432 |
| Ndufv1 | -0.9861 | 1.0133 | -0.0272 |
| Ndufa2 | -0.7011 | 1.1451 | -0.4441 |
| Vdac1 | -0.7322 | 1.1393 | -0.4071 |
| Ndufs1 | -0.7217 | 1.1415 | -0.4198 |
| Ndufa3 | -0.7154 | 1.1427 | -0.4273 |
| Ndufs2 | -0.5448 | 1.1541 | -0.6093 |
| Vdac2 | -0.5533 | 1.1544 | -0.6011 |
| Atp5f1 | -0.5588 | 1.1545 | -0.5957 |
| Ndufa4 | -0.6502 | 1.1515 | -0.5013 |
| Ndufb3 | -0.6570 | 1.1509 | -0.4938 |
| Ndufa11 | -0.6294 | 1.1531 | -0.5237 |
| Cox4i1 | -0.6244 | 1.1534 | -0.5289 |
| Cpt1b | -0.6269 | 1.1532 | -0.5264 |
| Pdha1 | -0.6062 | 1.1542 | -0.5480 |
| Cox7a1 | -0.5911 | 1.1546 | -0.5635 |
| Atp5a1 | 0.7083 | 0.4356 | -1.1439 |
| Ndufb11 | -0.0502 | 1.0242 | -0.9740 |
| Ndufb4 | -0.1442 | 1.0643 | -0.9201 |
| Ndufs3 | -0.1573 | 1.0693 | -0.9120 |
| Aifm1 | -0.3435 | 1.1265 | -0.7830 |
| Cox6a2 | -0.4865 | 1.1502 | -0.6636 |
| Aco2 | -0.4305 | 1.1431 | -0.7127 |
| Psmb6 | -0.7314 | -0.4082 | 1.1395 |
| Cul1 | -1.0892 | 0.8766 | 0.2127 |
| Psmc5 | 0.6104 | 0.5436 | -1.1541 |
| Uba1 | -0.5993 | 1.1544 | -0.5551 |
| Hspb1 | -0.4976 | 1.1512 | -0.6536 |
| Hsp90b1 | -0.4093 | 1.1397 | -0.7304 |
| Cryab | -0.4339 | 1.1437 | -0.7098 |
| Dnajc2 | 0.0033 | 0.9983 | -1.0017 |
| Hsp90ab1 | -0.1828 | 1.0788 | -0.8960 |
| Psmd2 | -0.3174 | 1.1202 | -0.8028 |
| Usp4 | 1.1073 | -0.2702 | -0.8371 |
| Psmb7 | 1.1226 | -0.3273 | -0.7954 |
| Hspb6 | 1.1256 | -0.3395 | -0.7860 |
| Psmd4 | 1.1438 | -0.7091 | -0.4347 |
| Ube2l3 | 1.1521 | -0.5091 | -0.6430 |
| Uchl3 | 1.1547 | -0.5720 | -0.5827 |
| Psma5 | 1.1020 | -0.8497 | -0.2522 |
| Hspb7 | 1.1107 | -0.8287 | -0.2821 |
| Hspa9 | 1.1130 | -0.8228 | -0.2902 |
| Usp12 | 1.1124 | -0.8243 | -0.2881 |
| Hspe1 | 0.9963 | -1.0036 | 0.0073 |
| Uchl1 | 1.0091 | -0.9907 | -0.0184 |
| Usp5 | 1.0669 | -0.9159 | -0.1511 |
| Psme1 | 1.0282 | -0.9692 | -0.0589 |
| Dnajc6 | 1.0457 | -0.9470 | -0.0987 |
| Psmb1 | 0.8922 | -1.0809 | 0.1887 |
| Psma6 | 0.8817 | -1.0866 | 0.2049 |
| Psma2 | 0.9657 | -1.0311 | 0.0653 |
| Skp1 | 0.9384 | -1.0519 | 0.1136 |
| Hspa5 | 0.9301 | -1.0576 | 0.1275 |
| Thop1 | 0.9290 | -1.0584 | 0.1293 |
| Dnajb8 | 0.5590 | -1.1545 | 0.5955 |
| Stub1 | 0.6039 | -1.1543 | 0.5503 |
| Hspa8 | 0.8198 | -1.1141 | 0.2943 |
| Dnajb5 | 0.7729 | -1.1294 | 0.3564 |
| B2m | 0.7892 | -1.1246 | 0.3354 |
| Hspb2 | 0.7880 | -1.1250 | 0.3370 |
| Ttn | 0.3811 | -1.1345 | 0.7534 |
| Cfl1 | 0.3627 | -1.1307 | 0.7680 |
| Arhgef6 | 0.3541 | -1.1289 | 0.7747 |
| Arpc1a | 0.7342 | -1.1389 | 0.4047 |
| Ppp1cb | 0.6140 | -1.1539 | 0.5399 |
| Pak7 | 0.6618 | -1.1504 | 0.4886 |
| Itga3 | 0.8509 | -1.1014 | 0.2505 |
| Flna | 0.8269 | -1.1114 | 0.2845 |
| Mylpf | 0.9623 | -1.0339 | 0.0716 |
| Myl4 | 0.9441 | -1.0478 | 0.1037 |
| Cfl2 | 0.9265 | -1.0601 | 0.1335 |
| Myl6 | 1.0284 | -0.9689 | -0.0595 |
| Kng1 | 1.0705 | -0.9101 | -0.1604 |
| Myl2 | 1.0562 | -0.9322 | -0.1241 |
| Myl9 | 1.0451 | -0.9477 | -0.0974 |
| Myl1 | 1.0976 | -0.8593 | -0.2383 |
| Myl3 | 1.0939 | -0.8671 | -0.2268 |
| Irs1 | 1.1177 | -0.8099 | -0.3079 |
| Kl | 1.1129 | -0.8231 | -0.2897 |
| Myl7 | 1.1128 | -0.8234 | -0.2894 |
| Gsn | 0.4857 | 0.6644 | -1.1501 |
| Cyfip2 | 0.6806 | 0.4675 | -1.1481 |
| Tln1 | 0.2144 | 0.8754 | -1.0898 |
| Msn | -0.0808 | 1.0379 | -0.9572 |
| Actn1 | -0.1698 | 1.0740 | -0.9042 |
| Myh7 | -1.0578 | 0.9299 | 0.1279 |
| Myh6 | -0.9380 | 1.0522 | -0.1142 |
| Actn2 | -0.9424 | 1.0491 | -0.1067 |
| Ptk2 | -0.9661 | 1.0308 | -0.0647 |
| Tln2 | -0.9642 | 1.0323 | -0.0682 |
| Fgf1 | -0.8179 | -0.2970 | 1.1149 |
| Apc2 | -1.1427 | 0.4276 | 0.7151 |
| Pygm | -0.4900 | 1.1505 | -0.6605 |
| Gnai2 | -0.1961 | 1.0835 | -0.8874 |
| Pdia3 | -0.1291 | 1.0583 | -0.9292 |
| Chp1 | -1.1318 | 0.3676 | 0.7642 |
| Ryr2 | -0.9740 | 1.0241 | -0.0502 |
| Flnc | -0.9748 | 1.0234 | -0.0485 |
| Prkaca | -0.8174 | 1.1150 | -0.2976 |
| Dusp3 | -0.9198 | 1.0644 | -0.1446 |
| Pygb | -0.8727 | 1.0912 | -0.2184 |
| Ywhaq | 0.6942 | -1.1462 | 0.4520 |
| Nfkb2 | 0.1658 | -1.0725 | 0.9068 |
| Akap12 | 0.4875 | -1.1503 | 0.6627 |
| Tnni3 | 0.9192 | -1.0648 | 0.1457 |
| Pgp | 0.9184 | -1.0654 | 0.1470 |
| Prkag1 | 0.9842 | -1.0151 | 0.0310 |
| Cnga2 | 1.0888 | -0.2114 | -0.8774 |
| Ywhaz | 1.0319 | -0.9647 | -0.0672 |
| Ywhae | 1.0646 | -0.9195 | -0.1451 |
| Ppp1r11 | 1.1406 | -0.7259 | -0.4148 |
| Ywhag | 1.0967 | -0.8613 | -0.2354 |
| Ppp2r1a | -0.3212 | 1.1211 | -0.7999 |
| Ilk | -1.1416 | 0.4205 | 0.7211 |
| Dsp | -1.0780 | 0.8974 | 0.1806 |
| Vim | -0.7912 | 1.1240 | -0.3328 |
| Parva | -0.9075 | 1.0721 | -0.1646 |
| Ppp2r5a | -0.9421 | 1.0493 | -0.1072 |
| Vcl | 0.6151 | -1.1539 | 0.5388 |
| Naca | 1.0676 | -0.9148 | -0.1528 |
| Fblim1 | 1.0769 | -0.8993 | -0.1776 |
| Sema6a | 0.5285 | -1.1534 | 0.6249 |
| Dpysl2 | 0.8531 | -1.1005 | 0.2474 |
| Gli2 | 1.1088 | -0.8335 | -0.2753 |
| Tuba8 | -0.9347 | 1.0545 | -0.1199 |
| Adam9 | -0.6766 | -0.4721 | 1.1486 |
| Ablim1 | -1.1469 | 0.6894 | 0.4575 |
| Cttn | 1.1107 | -0.8287 | -0.2820 |
| Zyx | 1.0727 | -0.9065 | -0.1661 |
| Itga2b | 0.4554 | -1.1466 | 0.6913 |
| Capn7 | 1.0230 | -0.0478 | -0.9753 |
| Itgav | -0.8239 | 1.1126 | -0.2887 |
| Capn2 | -1.1053 | 0.8420 | 0.2633 |
| Arf5 | -1.0488 | 0.9428 | 0.1060 |
| Clip1 | -0.9842 | 1.0151 | -0.0309 |
| Des | -0.6274 | 1.1532 | -0.5258 |
| Stmn1 | 1.0765 | -0.9000 | -0.1765 |
| Cdh13 | 1.1289 | -0.7747 | -0.3542 |
| Pard6a | 0.6942 | -1.1462 | 0.4520 |
| Atp2a2 | -0.8144 | 1.1161 | -0.3018 |
| Letm1 | -0.9762 | 1.0222 | -0.0460 |
| Casq2 | 0.8923 | 0.1886 | -1.0809 |
| Tpm3 | 0.5009 | 0.6505 | -1.1515 |
| Tnnt2 | 0.9665 | -1.0304 | 0.0639 |
| Calr | 1.1541 | -0.6092 | -0.5449 |
| Tpm2 | 1.0291 | -0.9681 | -0.0611 |
| Tnnc1 | 1.0702 | -0.9106 | -0.1596 |
| Tpm1 | 1.0791 | -0.8955 | -0.1836 |
| Tpm4 | 1.0810 | -0.8920 | -0.1890 |
| Lamtor3 | 0.7099 | -1.1436 | 0.4337 |
| Eif4ebp1 | 0.9355 | -1.0540 | 0.1185 |
| Mos | 1.1092 | -0.8327 | -0.2765 |

Supporting Table 3. IPA Categories and gene names from male heatmap in Figure 2B.

|  |  | **z-scores** | | |
| --- | --- | --- | --- | --- |
| **IPA Category** | **Gene ID** | **Control** | **Rapamycin** | **Persistence** |
| Mitochondrial Dysfunction & OxPhos | Atp5c1 | -0.0888 | -0.9526 | 1.0414 |
|  | Ndufab1 | 0.5678 | -1.1546 | 0.5869 |
|  | Ndufs4 | 0.2629 | -1.1052 | 0.8423 |
|  | Snca | 0.8572 | -1.0986 | 0.2415 |
|  | Prdx5 | 0.7841 | -1.1261 | 0.3420 |
|  | Sod2 | 0.9887 | -1.0109 | 0.0222 |
|  | Ndufs6 | 0.9958 | -1.0041 | 0.0083 |
|  | Atp5j | 0.9633 | -1.0330 | 0.0697 |
|  | Atp5b | 0.9307 | -1.0573 | 0.1266 |
|  | Txn2 | 0.9433 | -1.0484 | 0.1051 |
|  | Cox5a | 1.1408 | -0.7253 | -0.4154 |
|  | Ndufv3 | 1.1499 | -0.6658 | -0.4841 |
|  | Prdx3 | 1.0558 | -0.9328 | -0.1229 |
|  | Ndufv2 | 1.0650 | -0.9189 | -0.1462 |
|  | Atp5d | 1.1177 | -0.8100 | -0.3077 |
|  | Atp5e | 1.1044 | -0.8440 | -0.2604 |
|  | Xdh | 1.1083 | -0.8348 | -0.2735 |
|  | Cyc1 | -0.7916 | 1.1238 | -0.3322 |
|  | Uqcrc1 | -0.8158 | 1.1156 | -0.2998 |
|  | Ndufa9 | -0.8243 | 1.1124 | -0.2881 |
|  | Ndufa10 | -0.8638 | 1.0955 | -0.2317 |
|  | Cox7a2l | -0.8482 | 1.1026 | -0.2545 |
|  | Park7 | -0.8534 | 1.1003 | -0.2469 |
|  | Ndufb5 | -0.8893 | 1.0825 | -0.1932 |
|  | Ndufa8 | -0.9054 | 1.0733 | -0.1680 |
|  | Mt-Cyb | -1.0348 | 0.9612 | 0.0736 |
|  | Ndufb8 | -1.0347 | 0.9613 | 0.0734 |
|  | Ndufa1 | -1.0931 | 0.8688 | 0.2243 |
|  | Cox7b | -1.0605 | 0.9258 | 0.1347 |
|  | Sdha | -1.0624 | 0.9230 | 0.1393 |
|  | Ndufb6 | -0.9358 | 1.0537 | -0.1179 |
|  | Ndufa12 | -0.9429 | 1.0487 | -0.1058 |
|  | Fis1 | -0.9456 | 1.0467 | -0.1012 |
|  | Maob | -1.0114 | 0.9882 | 0.0231 |
|  | Ndufb7 | -0.9951 | 1.0048 | -0.0098 |
|  | Ogdh | -0.9777 | 1.0209 | -0.0432 |
|  | Ndufv1 | -0.9861 | 1.0133 | -0.0272 |
|  | Ndufa2 | -0.7011 | 1.1451 | -0.4441 |
|  | Vdac1 | -0.7322 | 1.1393 | -0.4071 |
|  | Ndufs1 | -0.7217 | 1.1415 | -0.4198 |
|  | Ndufa3 | -0.7154 | 1.1427 | -0.4273 |
|  | Ndufs2 | -0.5448 | 1.1541 | -0.6093 |
|  | Vdac2 | -0.5533 | 1.1544 | -0.6011 |
|  | Atp5f1 | -0.5588 | 1.1545 | -0.5957 |
|  | Ndufa4 | -0.6502 | 1.1515 | -0.5013 |
|  | Ndufb3 | -0.6570 | 1.1509 | -0.4938 |
|  | Ndufa11 | -0.6294 | 1.1531 | -0.5237 |
|  | Cox4i1 | -0.6244 | 1.1534 | -0.5289 |
|  | Cpt1b | -0.6269 | 1.1532 | -0.5264 |
|  | Pdha1 | -0.6062 | 1.1542 | -0.5480 |
|  | Cox7a1 | -0.5911 | 1.1546 | -0.5635 |
|  | Atp5a1 | 0.7083 | 0.4356 | -1.1439 |
|  | Ndufb11 | -0.0502 | 1.0242 | -0.9740 |
|  | Ndufb4 | -0.1442 | 1.0643 | -0.9201 |
|  | Ndufs3 | -0.1573 | 1.0693 | -0.9120 |
|  | Aifm1 | -0.3435 | 1.1265 | -0.7830 |
|  | Cox6a2 | -0.4865 | 1.1502 | -0.6636 |
|  | Aco2 | -0.4305 | 1.1431 | -0.7127 |
| Protein Ubiquitiniation Pathway | Psmb6 | -0.7314 | -0.4082 | 1.1395 |
|  | Cul1 | -1.0892 | 0.8766 | 0.2127 |
|  | Psmc5 | 0.6104 | 0.5436 | -1.1541 |
|  | Uba1 | -0.5993 | 1.1544 | -0.5551 |
|  | Hspb1 | -0.4976 | 1.1512 | -0.6536 |
|  | Hsp90b1 | -0.4093 | 1.1397 | -0.7304 |
|  | Cryab | -0.4339 | 1.1437 | -0.7098 |
|  | Dnajc2 | 0.0033 | 0.9983 | -1.0017 |
|  | Hsp90ab1 | -0.1828 | 1.0788 | -0.8960 |
|  | Psmd2 | -0.3174 | 1.1202 | -0.8028 |
|  | Usp4 | 1.1073 | -0.2702 | -0.8371 |
|  | Psmb7 | 1.1226 | -0.3273 | -0.7954 |
|  | Hspb6 | 1.1256 | -0.3395 | -0.7860 |
|  | Psmd4 | 1.1438 | -0.7091 | -0.4347 |
|  | Ube2l3 | 1.1521 | -0.5091 | -0.6430 |
|  | Uchl3 | 1.1547 | -0.5720 | -0.5827 |
|  | Psma5 | 1.1020 | -0.8497 | -0.2522 |
|  | Hspb7 | 1.1107 | -0.8287 | -0.2821 |
|  | Hspa9 | 1.1130 | -0.8228 | -0.2902 |
|  | Usp12 | 1.1124 | -0.8243 | -0.2881 |
|  | Hspe1 | 0.9963 | -1.0036 | 0.0073 |
|  | Uchl1 | 1.0091 | -0.9907 | -0.0184 |
|  | Usp5 | 1.0669 | -0.9159 | -0.1511 |
|  | Psme1 | 1.0282 | -0.9692 | -0.0589 |
|  | Dnajc6 | 1.0457 | -0.9470 | -0.0987 |
|  | Psmb1 | 0.8922 | -1.0809 | 0.1887 |
|  | Psma6 | 0.8817 | -1.0866 | 0.2049 |
|  | Psma2 | 0.9657 | -1.0311 | 0.0653 |
|  | Skp1 | 0.9384 | -1.0519 | 0.1136 |
|  | Hspa5 | 0.9301 | -1.0576 | 0.1275 |
|  | Thop1 | 0.9290 | -1.0584 | 0.1293 |
|  | Dnajb8 | 0.5590 | -1.1545 | 0.5955 |
|  | Stub1 | 0.6039 | -1.1543 | 0.5503 |
|  | Hspa8 | 0.8198 | -1.1141 | 0.2943 |
|  | Dnajb5 | 0.7729 | -1.1294 | 0.3564 |
|  | B2m | 0.7892 | -1.1246 | 0.3354 |
|  | Hspb2 | 0.7880 | -1.1250 | 0.3370 |
| Actin Cytoskeleton Signaling | Ttn | 0.3811 | -1.1345 | 0.7534 |
|  | Cfl1 | 0.3627 | -1.1307 | 0.7680 |
|  | Arhgef6 | 0.3541 | -1.1289 | 0.7747 |
|  | Arpc1a | 0.7342 | -1.1389 | 0.4047 |
|  | Ppp1cb | 0.6140 | -1.1539 | 0.5399 |
|  | Pak7 | 0.6618 | -1.1504 | 0.4886 |
|  | Itga3 | 0.8509 | -1.1014 | 0.2505 |
|  | Flna | 0.8269 | -1.1114 | 0.2845 |
|  | Mylpf | 0.9623 | -1.0339 | 0.0716 |
|  | Myl4 | 0.9441 | -1.0478 | 0.1037 |
|  | Cfl2 | 0.9265 | -1.0601 | 0.1335 |
|  | Myl6 | 1.0284 | -0.9689 | -0.0595 |
|  | Kng1 | 1.0705 | -0.9101 | -0.1604 |
|  | Myl2 | 1.0562 | -0.9322 | -0.1241 |
|  | Myl9 | 1.0451 | -0.9477 | -0.0974 |
|  | Myl1 | 1.0976 | -0.8593 | -0.2383 |
|  | Myl3 | 1.0939 | -0.8671 | -0.2268 |
|  | Irs1 | 1.1177 | -0.8099 | -0.3079 |
|  | Kl | 1.1129 | -0.8231 | -0.2897 |
|  | Myl7 | 1.1128 | -0.8234 | -0.2894 |
|  | Gsn | 0.4857 | 0.6644 | -1.1501 |
|  | Cyfip2 | 0.6806 | 0.4675 | -1.1481 |
|  | Tln1 | 0.2144 | 0.8754 | -1.0898 |
|  | Msn | -0.0808 | 1.0379 | -0.9572 |
|  | Actn1 | -0.1698 | 1.0740 | -0.9042 |
|  | Myh7 | -1.0578 | 0.9299 | 0.1279 |
|  | Ttn | -0.8754 | 1.0898 | -0.2145 |
|  | Myh6 | -0.9380 | 1.0522 | -0.1142 |
|  | Actn2 | -0.9424 | 1.0491 | -0.1067 |
|  | Ttn | -0.9798 | 1.0191 | -0.0393 |
|  | Ttn | -0.9538 | 1.0405 | -0.0867 |
|  | Ptk2 | -0.9661 | 1.0308 | -0.0647 |
|  | Tln2 | -0.9642 | 1.0323 | -0.0682 |
|  | Fgf1 | -0.8179 | -0.2970 | 1.1149 |
|  | Apc2 | -1.1427 | 0.4276 | 0.7151 |
| Protein Kinase A Signaling | Pygm | -0.4900 | 1.1505 | -0.6605 |
|  | Gnai2 | -0.1961 | 1.0835 | -0.8874 |
|  | Pdia3 | -0.1291 | 1.0583 | -0.9292 |
|  | Chp1 | -1.1318 | 0.3676 | 0.7642 |
|  | Ttn | -0.9798 | 1.0191 | -0.0393 |
|  | Ryr2 | -0.9740 | 1.0241 | -0.0502 |
|  | Flnc | -0.9748 | 1.0234 | -0.0485 |
|  | Ptk2 | -0.9661 | 1.0308 | -0.0647 |
|  | Ttn | -0.9538 | 1.0405 | -0.0867 |
|  | Prkaca | -0.8174 | 1.1150 | -0.2976 |
|  | Dusp3 | -0.9198 | 1.0644 | -0.1446 |
|  | Pygb | -0.8727 | 1.0912 | -0.2184 |
|  | Ttn | -0.8754 | 1.0898 | -0.2145 |
|  | Flna | 0.8269 | -1.1114 | 0.2845 |
|  | Ppp1cb | 0.6140 | -1.1539 | 0.5399 |
|  | Ywhaq | 0.6942 | -1.1462 | 0.4520 |
|  | Nfkb2 | 0.1658 | -1.0725 | 0.9068 |
|  | Ttn | 0.3811 | -1.1345 | 0.7534 |
|  | Akap12 | 0.4875 | -1.1503 | 0.6627 |
|  | Tnni3 | 0.9192 | -1.0648 | 0.1457 |
|  | Pgp | 0.9184 | -1.0654 | 0.1470 |
|  | Prkag1 | 0.9842 | -1.0151 | 0.0310 |
|  | Myl4 | 0.9441 | -1.0478 | 0.1037 |
|  | Mylpf | 0.9623 | -1.0339 | 0.0716 |
|  | Cnga2 | 1.0888 | -0.2114 | -0.8774 |
|  | Ywhaz | 1.0319 | -0.9647 | -0.0672 |
|  | Myl6 | 1.0284 | -0.9689 | -0.0595 |
|  | Myl9 | 1.0451 | -0.9477 | -0.0974 |
|  | Myl2 | 1.0562 | -0.9322 | -0.1241 |
|  | Ywhae | 1.0646 | -0.9195 | -0.1451 |
|  | Ppp1r11 | 1.1406 | -0.7259 | -0.4148 |
|  | Myl7 | 1.1128 | -0.8234 | -0.2894 |
|  | Myl3 | 1.0939 | -0.8671 | -0.2268 |
|  | Myl1 | 1.0976 | -0.8593 | -0.2383 |
|  | Ywhag | 1.0967 | -0.8613 | -0.2354 |
| ILK Signaling | Ppp2r1a | -0.3212 | 1.1211 | -0.7999 |
|  | Actn1 | -0.1698 | 1.0740 | -0.9042 |
|  | Ilk | -1.1416 | 0.4205 | 0.7211 |
|  | Dsp | -1.0780 | 0.8974 | 0.1806 |
|  | Myh7 | -1.0578 | 0.9299 | 0.1279 |
|  | Vim | -0.7912 | 1.1240 | -0.3328 |
|  | Ptk2 | -0.9661 | 1.0308 | -0.0647 |
|  | Flnc | -0.9748 | 1.0234 | -0.0485 |
|  | Parva | -0.9075 | 1.0721 | -0.1646 |
|  | Myh6 | -0.9380 | 1.0522 | -0.1142 |
|  | Ppp2r5a | -0.9421 | 1.0493 | -0.1072 |
|  | Actn2 | -0.9424 | 1.0491 | -0.1067 |
|  | Vcl | 0.6151 | -1.1539 | 0.5388 |
|  | Nfkb2 | 0.1658 | -1.0725 | 0.9068 |
|  | Cfl1 | 0.3627 | -1.1307 | 0.7680 |
|  | Arhgef6 | 0.3541 | -1.1289 | 0.7747 |
|  | Flna | 0.8269 | -1.1114 | 0.2845 |
|  | Ilk | 0.9947 | -1.0053 | 0.0106 |
|  | Myl4 | 0.9441 | -1.0478 | 0.1037 |
|  | Cfl2 | 0.9265 | -1.0601 | 0.1335 |
|  | Irs1 | 1.1177 | -0.8099 | -0.3079 |
|  | Kl | 1.1129 | -0.8231 | -0.2897 |
|  | Myl7 | 1.1128 | -0.8234 | -0.2894 |
|  | Myl6 | 1.0284 | -0.9689 | -0.0595 |
|  | Myl2 | 1.0562 | -0.9322 | -0.1241 |
|  | Myl9 | 1.0451 | -0.9477 | -0.0974 |
|  | Myl1 | 1.0976 | -0.8593 | -0.2383 |
|  | Myl3 | 1.0939 | -0.8671 | -0.2268 |
|  | Naca | 1.0676 | -0.9148 | -0.1528 |
|  | Fblim1 | 1.0769 | -0.8993 | -0.1776 |
| Axonal Guidance Signaling | Cfl1 | 0.3627 | -1.1307 | 0.7680 |
|  | Arhgef6 | 0.3541 | -1.1289 | 0.7747 |
|  | Sema6a | 0.5285 | -1.1534 | 0.6249 |
|  | Pak7 | 0.6618 | -1.1504 | 0.4886 |
|  | Arpc1a | 0.7342 | -1.1389 | 0.4047 |
|  | Dpysl2 | 0.8531 | -1.1005 | 0.2474 |
|  | Itga3 | 0.8509 | -1.1014 | 0.2505 |
|  | Myl4 | 0.9441 | -1.0478 | 0.1037 |
|  | Cfl2 | 0.9265 | -1.0601 | 0.1335 |
|  | Prkag1 | 0.9842 | -1.0151 | 0.0310 |
|  | Mylpf | 0.9623 | -1.0339 | 0.0716 |
|  | Myl6 | 1.0284 | -0.9689 | -0.0595 |
|  | Myl2 | 1.0562 | -0.9322 | -0.1241 |
|  | Myl9 | 1.0451 | -0.9477 | -0.0974 |
|  | Myl1 | 1.0976 | -0.8593 | -0.2383 |
|  | Myl3 | 1.0939 | -0.8671 | -0.2268 |
|  | Irs1 | 1.1177 | -0.8099 | -0.3079 |
|  | Gli2 | 1.1088 | -0.8335 | -0.2753 |
|  | Kl | 1.1129 | -0.8231 | -0.2897 |
|  | Myl7 | 1.1128 | -0.8234 | -0.2894 |
|  | Gli2 | 0.4287 | 0.7142 | -1.1429 |
|  | Gnai2 | -0.1961 | 1.0835 | -0.8874 |
|  | Pdia3 | -0.1291 | 1.0583 | -0.9292 |
|  | Prkaca | -0.8174 | 1.1150 | -0.2976 |
|  | Ptk2 | -0.9661 | 1.0308 | -0.0647 |
|  | Tuba8 | -0.9347 | 1.0545 | -0.1199 |
|  | Adam9 | -0.6766 | -0.4721 | 1.1486 |
|  | Chp1 | -1.1318 | 0.3676 | 0.7642 |
|  | Ablim1 | -1.1469 | 0.6894 | 0.4575 |
| Integrin Signaling | Irs1 | 1.1177 | -0.8099 | -0.3079 |
|  | Cttn | 1.1107 | -0.8287 | -0.2820 |
|  | Kl | 1.1129 | -0.8231 | -0.2897 |
|  | Myl7 | 1.1128 | -0.8234 | -0.2894 |
|  | Ilk | 0.9947 | -1.0053 | 0.0106 |
|  | Zyx | 1.0727 | -0.9065 | -0.1661 |
|  | Myl2 | 1.0562 | -0.9322 | -0.1241 |
|  | Myl9 | 1.0451 | -0.9477 | -0.0974 |
|  | Ttn | 0.3811 | -1.1345 | 0.7534 |
|  | Itga2b | 0.4554 | -1.1466 | 0.6913 |
|  | Pak7 | 0.6618 | -1.1504 | 0.4886 |
|  | Ppp1cb | 0.6140 | -1.1539 | 0.5399 |
|  | Vcl | 0.6151 | -1.1539 | 0.5388 |
|  | Itga3 | 0.8509 | -1.1014 | 0.2505 |
|  | Arpc1a | 0.7342 | -1.1389 | 0.4047 |
|  | Capn7 | 1.0230 | -0.0478 | -0.9753 |
|  | Actn1 | -0.1698 | 1.0740 | -0.9042 |
|  | Gsn | 0.4857 | 0.6644 | -1.1501 |
|  | Tln1 | 0.2144 | 0.8754 | -1.0898 |
|  | Ttn | -0.9538 | 1.0405 | -0.0867 |
|  | Actn2 | -0.9424 | 1.0491 | -0.1067 |
|  | Ttn | -0.9798 | 1.0191 | -0.0393 |
|  | Ptk2 | -0.9661 | 1.0308 | -0.0647 |
|  | Tln2 | -0.9642 | 1.0323 | -0.0682 |
|  | Itgav | -0.8239 | 1.1126 | -0.2887 |
|  | Ttn | -0.8754 | 1.0898 | -0.2145 |
|  | Parva | -0.9075 | 1.0721 | -0.1646 |
|  | Ilk | -1.1416 | 0.4205 | 0.7211 |
|  | Capn2 | -1.1053 | 0.8420 | 0.2633 |
|  | Arf5 | -1.0488 | 0.9428 | 0.1060 |
| Signaling by Rho Family GTPases | Gnai2 | -0.1961 | 1.0835 | -0.8874 |
|  | Msn | -0.0808 | 1.0379 | -0.9572 |
|  | Clip1 | -0.9842 | 1.0151 | -0.0309 |
|  | Ptk2 | -0.9661 | 1.0308 | -0.0647 |
|  | Vim | -0.7912 | 1.1240 | -0.3328 |
|  | Des | -0.6274 | 1.1532 | -0.5258 |
|  | Myl6 | 1.0284 | -0.9689 | -0.0595 |
|  | Clip1 | 1.0180 | -0.9810 | -0.0370 |
|  | Myl2 | 1.0562 | -0.9322 | -0.1241 |
|  | Myl9 | 1.0451 | -0.9477 | -0.0974 |
|  | Stmn1 | 1.0765 | -0.9000 | -0.1765 |
|  | Myl1 | 1.0976 | -0.8593 | -0.2383 |
|  | Myl3 | 1.0939 | -0.8671 | -0.2268 |
|  | Cdh13 | 1.1289 | -0.7747 | -0.3542 |
|  | Irs1 | 1.1177 | -0.8099 | -0.3079 |
|  | Kl | 1.1129 | -0.8231 | -0.2897 |
|  | Myl7 | 1.1128 | -0.8234 | -0.2894 |
|  | Nfkb2 | 0.1658 | -1.0725 | 0.9068 |
|  | Cfl1 | 0.3627 | -1.1307 | 0.7680 |
|  | Arhgef6 | 0.3541 | -1.1289 | 0.7747 |
|  | Arpc1a | 0.7342 | -1.1389 | 0.4047 |
|  | Pak7 | 0.6618 | -1.1504 | 0.4886 |
|  | Pard6a | 0.6942 | -1.1462 | 0.4520 |
|  | Itga3 | 0.8509 | -1.1014 | 0.2505 |
|  | Mylpf | 0.9623 | -1.0339 | 0.0716 |
|  | Cfl2 | 0.9265 | -1.0601 | 0.1335 |
|  | Clip1 | 0.9388 | -1.0517 | 0.1129 |
|  | Myl4 | 0.9441 | -1.0478 | 0.1037 |
| Calcium Signaling | Chp1 | -1.1318 | 0.3676 | 0.7642 |
|  | Atp2a2 | -0.8144 | 1.1161 | -0.3018 |
|  | Prkaca | -0.8174 | 1.1150 | -0.2976 |
|  | Myh7 | -1.0578 | 0.9299 | 0.1279 |
|  | Myh6 | -0.9380 | 1.0522 | -0.1142 |
|  | Ryr2 | -0.9740 | 1.0241 | -0.0502 |
|  | Letm1 | -0.9762 | 1.0222 | -0.0460 |
|  | Casq2 | 0.8923 | 0.1886 | -1.0809 |
|  | Tpm3 | 0.5009 | 0.6505 | -1.1515 |
|  | Prkag1 | 0.9842 | -1.0151 | 0.0310 |
|  | Tnnt2 | 0.9665 | -1.0304 | 0.0639 |
|  | Myl4 | 0.9441 | -1.0478 | 0.1037 |
|  | Tnni3 | 0.9192 | -1.0648 | 0.1457 |
|  | Calr | 1.1541 | -0.6092 | -0.5449 |
|  | Tpm2 | 1.0291 | -0.9681 | -0.0611 |
|  | Myl6 | 1.0284 | -0.9689 | -0.0595 |
|  | Myl2 | 1.0562 | -0.9322 | -0.1241 |
|  | Myl9 | 1.0451 | -0.9477 | -0.0974 |
|  | Tnnc1 | 1.0702 | -0.9106 | -0.1596 |
|  | Tpm1 | 1.0791 | -0.8955 | -0.1836 |
|  | Tpm4 | 1.0810 | -0.8920 | -0.1890 |
|  | Myl7 | 1.1128 | -0.8234 | -0.2894 |
|  | Myl1 | 1.0976 | -0.8593 | -0.2383 |
|  | Myl3 | 1.0939 | -0.8671 | -0.2268 |
| ERKMAPK Signaling | Prkaca | -0.8174 | 1.1150 | -0.2976 |
|  | Ppp2r5a | -0.9421 | 1.0493 | -0.1072 |
|  | Ptk2 | -0.9661 | 1.0308 | -0.0647 |
|  | Tln2 | -0.9642 | 1.0323 | -0.0682 |
|  | Tln1 | 0.2144 | 0.8754 | -1.0898 |
|  | Hspb1 | -0.4976 | 1.1512 | -0.6536 |
|  | Ppp2r1a | -0.3212 | 1.1211 | -0.7999 |
|  | Itga3 | 0.8509 | -1.1014 | 0.2505 |
|  | Hspb2 | 0.7880 | -1.1250 | 0.3370 |
|  | Lamtor3 | 0.7099 | -1.1436 | 0.4337 |
|  | Ywhaq | 0.6942 | -1.1462 | 0.4520 |
|  | Ppp1cb | 0.6140 | -1.1539 | 0.5399 |
|  | Pak7 | 0.6618 | -1.1504 | 0.4886 |
|  | Ywhaz | 1.0319 | -0.9647 | -0.0672 |
|  | Prkag1 | 0.9842 | -1.0151 | 0.0310 |
|  | Eif4ebp1 | 0.9355 | -1.0540 | 0.1185 |
|  | Ppp1r11 | 1.1406 | -0.7259 | -0.4148 |
|  | Ywhag | 1.0967 | -0.8613 | -0.2354 |
|  | Irs1 | 1.1177 | -0.8099 | -0.3079 |
|  | Kl | 1.1129 | -0.8231 | -0.2897 |
|  | Mos | 1.1092 | -0.8327 | -0.2765 |
|  | Hspb7 | 1.1107 | -0.8287 | -0.2821 |

Supporting Table 4. IPA Categories and gene names from female heatmap in Figure 2B.

|  |  | z-scores | | |
| --- | --- | --- | --- | --- |
| IPA Category | Gene ID | Control | Rapamycin | Persistence |
| Mitochondrial Dysfunction & OxPhos | Atp5f1 | -1.04066 | 0.087008 | 0.953653 |
|  | Ndufs2 | -1.13136 | 0.365655 | 0.765709 |
|  | Vdac1 | -1.14935 | 0.478518 | 0.670832 |
|  | Ndufb4 | -1.15066 | 0.491758 | 0.658903 |
|  | Ndufs1 | -1.15429 | 0.550415 | 0.603873 |
|  | Ndufb8 | -1.15421 | 0.547975 | 0.606235 |
|  | Ogdh | -1.15313 | 0.524362 | 0.628764 |
|  | Ndufa2 | -1.15351 | 0.531371 | 0.62214 |
|  | Atp5o | -0.86724 | 1.093862 | -0.22662 |
|  | Cpt1b | -1.0016 | 0.99839 | 0.003212 |
|  | Sdhd | -1.07977 | 0.89424 | 0.185534 |
|  | Uqcrc1 | -1.04439 | 0.948741 | 0.095645 |
|  | Ndufb11 | -1.14839 | 0.678624 | 0.469763 |
|  | Cox7a1 | -1.14482 | 0.70296 | 0.441859 |
|  | Cox6a2 | -1.147 | 0.688825 | 0.458171 |
|  | Vdac2 | -1.14627 | 0.693776 | 0.452491 |
|  | Vdac3 | -1.10141 | 0.850986 | 0.250427 |
|  | Cyc1 | -1.11147 | 0.826811 | 0.284654 |
|  | Ndufa9 | -1.10972 | 0.831258 | 0.278458 |
|  | Ndufa4 | -1.13598 | 0.747326 | 0.388654 |
|  | Cox6c | -1.13974 | 0.730339 | 0.409397 |
|  | Ndufa3 | -1.12344 | 0.792818 | 0.330626 |
|  | Ndufb7 | -1.13296 | 0.759615 | 0.373346 |
|  | Ndufa10 | -1.13125 | 0.766152 | 0.365093 |
|  | Ndufa8 | -1.13023 | 0.769878 | 0.360356 |
|  | Cox5a | 0.401191 | 0.737106 | -1.1383 |
|  | Ndufab1 | -0.13311 | 1.059887 | -0.92678 |
|  | Txn2 | 1.065217 | -0.91859 | -0.14662 |
|  | Ndufv2 | 1.127022 | -0.78115 | -0.34587 |
|  | Atp5j | 0.912713 | 0.156192 | -1.06891 |
|  | Ndufs6 | 1.108418 | -0.27393 | -0.83449 |
|  | Ndufv3 | 1.091861 | -0.22054 | -0.87132 |
|  | Prdx3 | 1.143891 | -0.43543 | -0.70846 |
|  | Atp5d | 1.153806 | -0.53755 | -0.61625 |
| Protein Kinase A Signaling | Myl9 | 0.977543 | 0.043495 | -1.02104 |
|  | Myl1 | 1.029273 | -0.06137 | -0.9679 |
|  | Myl7 | 1.022234 | -0.04606 | -0.97617 |
|  | Myl3 | 1.087712 | -0.20821 | -0.87951 |
|  | Ppp1r7 | 1.09182 | -0.22042 | -0.8714 |
|  | Myl4 | 1.124814 | -0.33636 | -0.78845 |
|  | Myl2 | 1.108296 | -0.27351 | -0.83479 |
|  | Ywhag | 1.047627 | -0.94436 | -0.10327 |
|  | Akap12 | 1.083141 | -0.88813 | -0.19501 |
|  | Tnni3 | 1.15469 | -0.58153 | -0.57316 |
|  | Ywhah | 1.151676 | -0.64816 | -0.50351 |
|  | Ywhaz | 1.142211 | -0.42443 | -0.71778 |
|  | Pgp | 1.142346 | -0.42528 | -0.71706 |
|  | Ywhae | 1.147125 | -0.4592 | -0.68792 |
|  | Ppp1cb | 1.150012 | -0.48499 | -0.66503 |
|  | Myl6 | 1.151874 | -0.50602 | -0.64586 |
|  | Ctnnb1 | -0.1722 | 1.074918 | -0.90272 |
|  | Pygb | -0.83647 | 1.107612 | -0.27115 |
|  | Camk2d | -0.55667 | -0.59779 | 1.154456 |
|  | Ttn | -0.823 | -0.28994 | 1.112931 |
|  | Ttn | -1.12798 | 0.350127 | 0.777857 |
|  | Ryr2 | -1.10892 | 0.275669 | 0.83325 |
|  | Ttn | -1.1515 | 0.501293 | 0.650203 |
|  | Gnb2 | -1.15432 | 0.602733 | 0.55159 |
|  | Flnc | -1.15448 | 0.596674 | 0.557809 |
| ILK Signaling | Myl9 | 0.977543 | 0.043495 | -1.02104 |
|  | Naca | 1.016368 | -0.03358 | -0.98279 |
|  | Myl1 | 1.029273 | -0.06137 | -0.9679 |
|  | Myh9 | 1.024039 | -0.04995 | -0.97409 |
|  | Myl7 | 1.022234 | -0.04606 | -0.97617 |
|  | Myl4 | 1.124814 | -0.33636 | -0.78845 |
|  | Cfl1 | 1.133477 | -0.37589 | -0.75759 |
|  | Myl3 | 1.087712 | -0.20821 | -0.87951 |
|  | Myl2 | 1.108296 | -0.27351 | -0.83479 |
|  | Vim | 1.141986 | -0.71898 | -0.423 |
|  | Myl6 | 1.151874 | -0.50602 | -0.64586 |
|  | Ctnnb1 | -0.1722 | 1.074918 | -0.90272 |
|  | Actn2 | -1.13177 | 0.764175 | 0.367598 |
|  | Ppp2r1a | -1.14198 | 0.422952 | 0.719026 |
|  | Flnc | -1.15448 | 0.596674 | 0.557809 |
|  | Myh13 | 0.669363 | -1.14952 | 0.480159 |
|  | Acta1 | 0.998668 | -1.00133 | 0.002659 |
|  | Dsp | -0.86311 | -0.23273 | 1.095844 |
|  | Myh6 | -0.45815 | -0.68884 | 1.146993 |
|  | Myh7 | -0.13776 | -0.92398 | 1.061738 |
| Calcium Signaling | Calr | 0.891363 | 0.190011 | -1.08137 |
|  | Myl9 | 0.977543 | 0.043495 | -1.02104 |
|  | Myl1 | 1.029273 | -0.06137 | -0.9679 |
|  | Myh9 | 1.024039 | -0.04995 | -0.97409 |
|  | Myl7 | 1.022234 | -0.04606 | -0.97617 |
|  | Acta1 | 0.998668 | -1.00133 | 0.002659 |
|  | Tnni3 | 1.15469 | -0.58153 | -0.57316 |
|  | Tpm4 | 1.154637 | -0.56682 | -0.58782 |
|  | Tpm1 | 1.149395 | -0.47895 | -0.67045 |
|  | Myl6 | 1.151874 | -0.50602 | -0.64586 |
|  | Myl4 | 1.124814 | -0.33636 | -0.78845 |
|  | Tnnt2 | 1.131121 | -0.36451 | -0.76662 |
|  | Myl3 | 1.087712 | -0.20821 | -0.87951 |
|  | Myl2 | 1.108296 | -0.27351 | -0.83479 |
|  | Ryr2 | -1.10892 | 0.275669 | 0.83325 |
|  | Atp2a2 | -1.15193 | 0.506728 | 0.645202 |
|  | Myh7 | -0.13776 | -0.92398 | 1.061738 |
|  | Myh6 | -0.45815 | -0.68884 | 1.146993 |
|  | Camk2d | -0.55667 | -0.59779 | 1.154456 |
| Actin Cytoskeleton Signaling | Kng1 | 0.852184 | 0.248695 | -1.10088 |
|  | Myl9 | 0.977543 | 0.043495 | -1.02104 |
|  | Myl1 | 1.029273 | -0.06137 | -0.9679 |
|  | Myh9 | 1.024039 | -0.04995 | -0.97409 |
|  | Myl7 | 1.022234 | -0.04606 | -0.97617 |
|  | Ppp1cb | 1.150012 | -0.48499 | -0.66503 |
|  | Myl6 | 1.151874 | -0.50602 | -0.64586 |
|  | Myl4 | 1.124814 | -0.33636 | -0.78845 |
|  | Cfl1 | 1.133477 | -0.37589 | -0.75759 |
|  | Myl3 | 1.087712 | -0.20821 | -0.87951 |
|  | Myl2 | 1.108296 | -0.27351 | -0.83479 |
|  | Actn2 | -1.13177 | 0.764175 | 0.367598 |
|  | Vav1 | -1.15456 | 0.561862 | 0.592701 |
|  | Ttn | -1.1515 | 0.501293 | 0.650203 |
|  | Ttn | -1.12798 | 0.350127 | 0.777857 |
|  | Rac1 | -1.14166 | 0.420968 | 0.720692 |
|  | Myh13 | 0.669363 | -1.14952 | 0.480159 |
|  | Acta1 | 0.998668 | -1.00133 | 0.002659 |
|  | Ttn | -0.823 | -0.28994 | 1.112931 |
|  | Myh6 | -0.45815 | -0.68884 | 1.146993 |
|  | Myh7 | -0.13776 | -0.92398 | 1.061738 |
| Epithelial Adherens Junction Signaling | Clip1 | 0.274893 | 0.833803 | -1.1087 |
|  | Ctnnb1 | -0.1722 | 1.074918 | -0.90272 |
|  | Myl6 | 1.151874 | -0.50602 | -0.64586 |
|  | Clip1 | 1.143387 | -0.43205 | -0.71134 |
|  | Myl3 | 1.087712 | -0.20821 | -0.87951 |
|  | Myl4 | 1.124814 | -0.33636 | -0.78845 |
|  | Myl2 | 1.108296 | -0.27351 | -0.83479 |
|  | Clip1 | 0.850786 | 0.250715 | -1.1015 |
|  | Myl9 | 0.977543 | 0.043495 | -1.02104 |
|  | Myl1 | 1.029273 | -0.06137 | -0.9679 |
|  | Myh9 | 1.024039 | -0.04995 | -0.97409 |
|  | Myl7 | 1.022234 | -0.04606 | -0.97617 |
|  | Actn2 | -1.13177 | 0.764175 | 0.367598 |
|  | Jup | -1.08363 | 0.196394 | 0.887233 |
|  | Rac1 | -1.14166 | 0.420968 | 0.720692 |
|  | Sorbs1 | -1.13008 | 0.359641 | 0.770439 |
|  | Myh6 | -0.45815 | -0.68884 | 1.146993 |
|  | Sorbs1 | -0.29973 | -0.81586 | 1.115588 |
|  | Myh7 | -0.13776 | -0.92398 | 1.061738 |
|  | Myh13 | 0.669363 | -1.14952 | 0.480159 |
|  | Acta1 | 0.998668 | -1.00133 | 0.002659 |
| Signaling by Rho Family GTPases | Gnb2 | -1.15432 | 0.602733 | 0.55159 |
|  | Rac1 | -1.14166 | 0.420968 | 0.720692 |
|  | Clip1 | 0.274893 | 0.833803 | -1.1087 |
|  | Clip1 | 0.850786 | 0.250715 | -1.1015 |
|  | Myl9 | 0.977543 | 0.043495 | -1.02104 |
|  | Map3k10 | 1.047226 | -0.10232 | -0.94491 |
|  | Myl1 | 1.029273 | -0.06137 | -0.9679 |
|  | Myl7 | 1.022234 | -0.04606 | -0.97617 |
|  | Myl3 | 1.087712 | -0.20821 | -0.87951 |
|  | Myl2 | 1.108296 | -0.27351 | -0.83479 |
|  | Cdh13 | 1.104667 | -0.26116 | -0.84351 |
|  | Myl6 | 1.151874 | -0.50602 | -0.64586 |
|  | Myl4 | 1.124814 | -0.33636 | -0.78845 |
|  | Clip1 | 1.143387 | -0.43205 | -0.71134 |
|  | Cfl1 | 1.133477 | -0.37589 | -0.75759 |
|  | Arhgef17 | 1.13759 | -0.39728 | -0.74031 |
|  | Acta1 | 0.998668 | -1.00133 | 0.002659 |
|  | Vim | 1.141986 | -0.71898 | -0.423 |
| Tight Junction Signaling | Myl9 | 0.977543 | 0.043495 | -1.02104 |
|  | Myl1 | 1.029273 | -0.06137 | -0.9679 |
|  | Myh9 | 1.024039 | -0.04995 | -0.97409 |
|  | Myl7 | 1.022234 | -0.04606 | -0.97617 |
|  | Myl3 | 1.087712 | -0.20821 | -0.87951 |
|  | Myl4 | 1.124814 | -0.33636 | -0.78845 |
|  | Myl2 | 1.108296 | -0.27351 | -0.83479 |
|  | Sptan1 | 1.138395 | -0.40174 | -0.73666 |
|  | Myl6 | 1.151874 | -0.50602 | -0.64586 |
|  | Myh6 | -0.45815 | -0.68884 | 1.146993 |
|  | Myh7 | -0.13776 | -0.92398 | 1.061738 |
|  | Myh13 | 0.669363 | -1.14952 | 0.480159 |
|  | Acta1 | 0.998668 | -1.00133 | 0.002659 |
|  | Ctnnb1 | -0.1722 | 1.074918 | -0.90272 |
|  | Rac1 | -1.14166 | 0.420968 | 0.720692 |
|  | Ppp2r1a | -1.14198 | 0.422952 | 0.719026 |
| RhoGDI Signaling | Gnb2 | -1.15432 | 0.602733 | 0.55159 |
|  | Rac1 | -1.14166 | 0.420968 | 0.720692 |
|  | Acta1 | 0.998668 | -1.00133 | 0.002659 |
|  | Arhgdib | 0.97072 | -1.02691 | 0.05619 |
|  | Myl3 | 1.087712 | -0.20821 | -0.87951 |
|  | Myl2 | 1.108296 | -0.27351 | -0.83479 |
|  | Cdh13 | 1.104667 | -0.26116 | -0.84351 |
|  | Myl6 | 1.151874 | -0.50602 | -0.64586 |
|  | Myl4 | 1.124814 | -0.33636 | -0.78845 |
|  | Cfl1 | 1.133477 | -0.37589 | -0.75759 |
|  | Arhgef17 | 1.13759 | -0.39728 | -0.74031 |
|  | Arhgdia | 0.755531 | 0.378462 | -1.13399 |
|  | Myl9 | 0.977543 | 0.043495 | -1.02104 |
|  | Myl1 | 1.029273 | -0.06137 | -0.9679 |
|  | Myl7 | 1.022234 | -0.04606 | -0.97617 |
| Cellular Effects of Sildenafil Viagra | Ppp1cb | 1.150012 | -0.48499 | -0.66503 |
|  | Myl6 | 1.151874 | -0.50602 | -0.64586 |
|  | Myl3 | 1.087712 | -0.20821 | -0.87951 |
|  | Myl4 | 1.124814 | -0.33636 | -0.78845 |
|  | Myl2 | 1.108296 | -0.27351 | -0.83479 |
|  | Nppa | 0.76706 | 0.363942 | -1.131 |
|  | Myl9 | 0.977543 | 0.043495 | -1.02104 |
|  | Myl1 | 1.029273 | -0.06137 | -0.9679 |
|  | Myh9 | 1.024039 | -0.04995 | -0.97409 |
|  | Myl7 | 1.022234 | -0.04606 | -0.97617 |
|  | Myh6 | -0.45815 | -0.68884 | 1.146993 |
|  | Myh7 | -0.13776 | -0.92398 | 1.061738 |
|  | Myh13 | 0.669363 | -1.14952 | 0.480159 |
|  | Acta1 | 0.998668 | -1.00133 | 0.002659 |

| **Average % Persistence per IPA Category** | | | | | | |
| --- | --- | --- | --- | --- | --- | --- |
| (includes proteins for which: -200 < % persistence < 200) | | | | | | |
|  | **females** | | | **males** | | |
| **IPA category** | **%** | **SD** | ***n*** | **%** | **SD** | ***n*** |
| actin cytoskeleton signaling | 116.6 | 54.1 | *11* | 39.1 | 31.1 | *24* |
| calcium signaling | 132.8 | 55.5 | *15* | 55.1 | 15.2 | *19* |
| ILK signaling | 119.6 | 56.4 | *12* | 42.5 | 37 | *26* |
| mito dysfunction & oxphos | 86.1 | 43.6 | *27* | 21.3 | 41.5 | *55* |
| protein kinase A signaling | 113.3 | 37.3 | *18* | 39.7 | 39.9 | *29* |
| protein ubiquitination pathway | 121.7 | 34.4 | *6* | 40.9 | 46.7 | *23* |

**Supporting Table 5. Average percent persistence per IPA category per sex.**

The top 5 IPA categories per sex, 6 altogether with two different between the sexes and four the same, are listed along with the average percent persistence of all proteins found in each category. The standard deviation (SD) and the number of proteins in each category (n) are also shown. The data were limited to proteins with percent persistence between -200% and 200% to eliminate outliers.

**
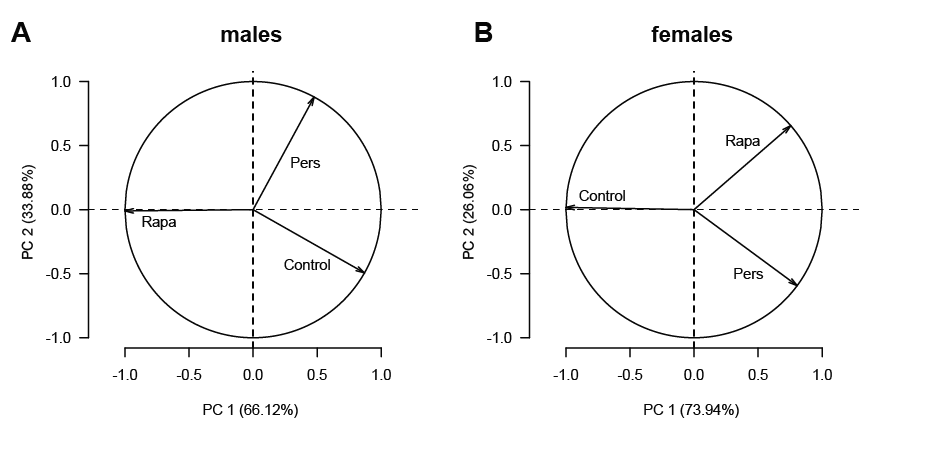
**

**Supporting Figure 2. Loading plots from principle component analysis.**

Shown are loading plots from PCA of male (A) and female (B) proteomic data from Figure 3A. If two vectors are close to each other, the variables are highly correlated. When the angle between vectors is 90, the variables are uncorrelated, and when the angle is large, they are anti-correlated. Comparisons are between untreated Control, continuous Rapamycin treatment, and Pers = Persistence 8 weeks after cessation of 8 week treatment.


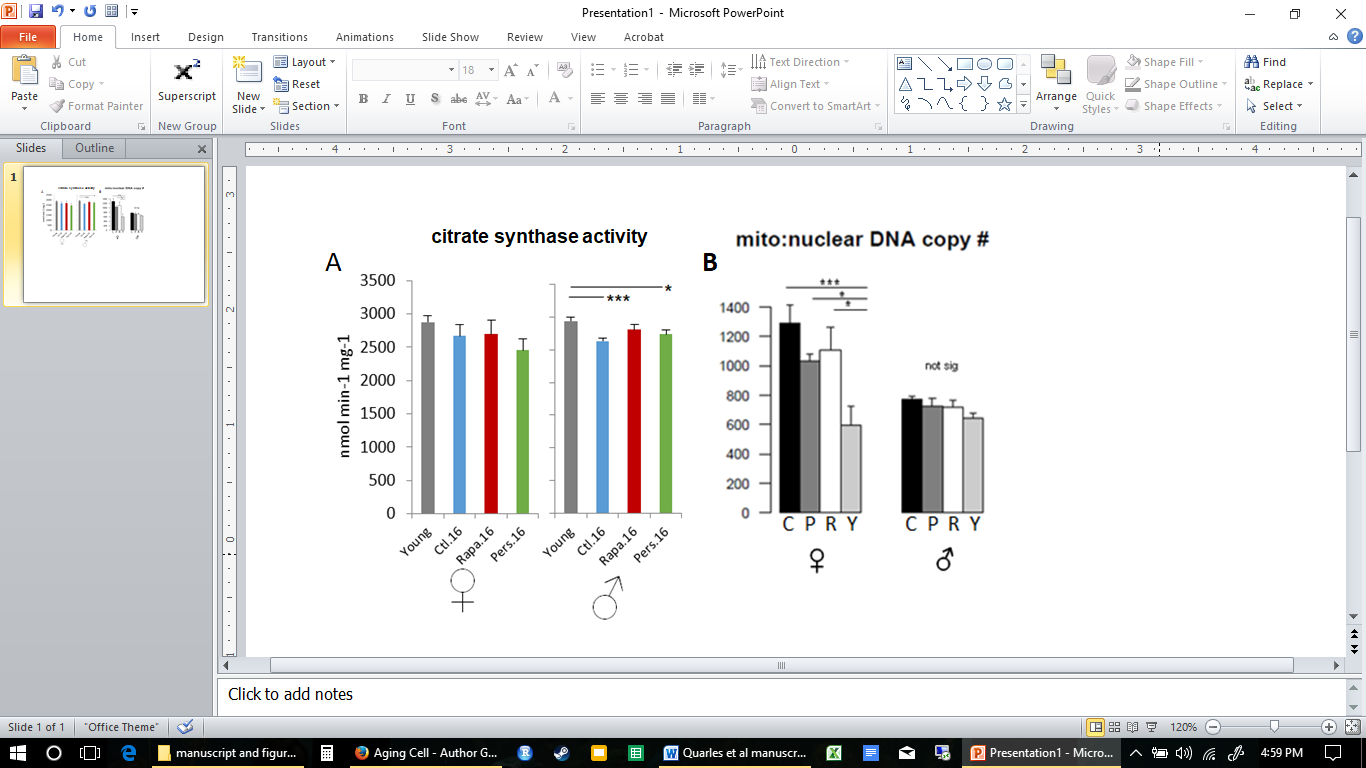


**Supporting Figure 3. Measures of mitochondrial content in both sexes.**

A) Citrate synthase activity per min per mg tissue. Left: females, right: males. Significance determined by ANOVA followed by Tukey post-hoc test. * p<0.05, **p<0.01, *** p<0.001. n per group: 6 ~ 15. Error bars are SEM. B) Aging in females, but not males, increases mitochondrial:nuclear DNA ratio. Bar charts for females (left) and males (right) show the ratio of mitochondrial:nuclear DNA ratio (mt-nd1:CYP1a1). Error bars are SEM. Significance was determined by one-way ANOVA with Tukey post hoc test. C = old control, P = old persistence, R = old rapa, Y = young mice. * p<0.05, **p<0.01, *** p<0.001. n per group: 7~8

The activity of the ETC complexes was determined in the context of the mitochondrial content, by normalizing to the citrate synthase (CS) activity of each sample. This is a commonly accepted method of accounting for differences in the mitochondrial content of a sample. There was a slight decrease in CS activity in the control and persistence groups compared to young animals in the males, but this difference did not noticeably alter the degree of significance found in the comparisons of ETC complex assays normalized to the CS activity. We also investigated another measure of mitochondrial content, mitochondrial:nuclear DNA ratio. In females, which generally had higher ratios of mito:nuclear DNA than males, there were significant increases in all old groups with age, but no significant differences between the old groups (control, rapamycin, persistence). In males, there were no detected differences due to aging or rapamycin. The CS activity data and the mito:nuclear DNA ratio data both show modest to no detectable differences between the old mice groups for either sex, leading to the conclusion that the mitochondrial content was similar between all old groups within each sex.

Supporting Figure 4. TCA cycle component abundances (next page)


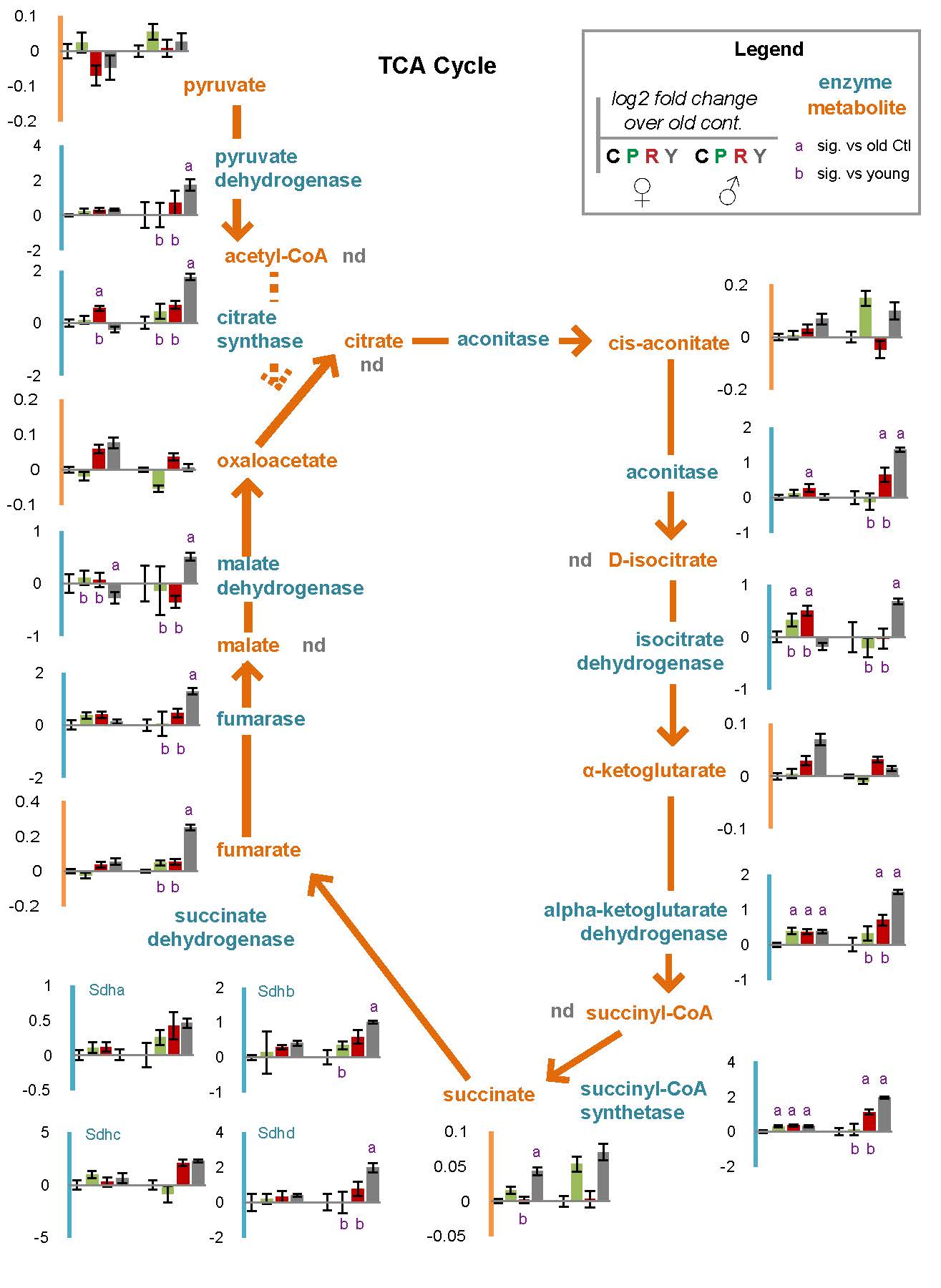


**Supporting Figure 4. Metabolomic and proteomic abundance changes in the TCA cycle.** This diagram shows the log2-fold-change from old control mice, in both sexes, in the enzymes (blue) and metabolites (orange) present in the TCA cycle. nd – no data. Sdh(a/b/c/d) – succinate dehydrogenase a/b/c/d respectively. Statistical significance by one-way ANOVA followed by Tukey post-hoc test: group was significantly different compared to old control (a) or group was significantly different from young control (b) of the same sex.

Supporting Figure 5. Glycolysis component abundances


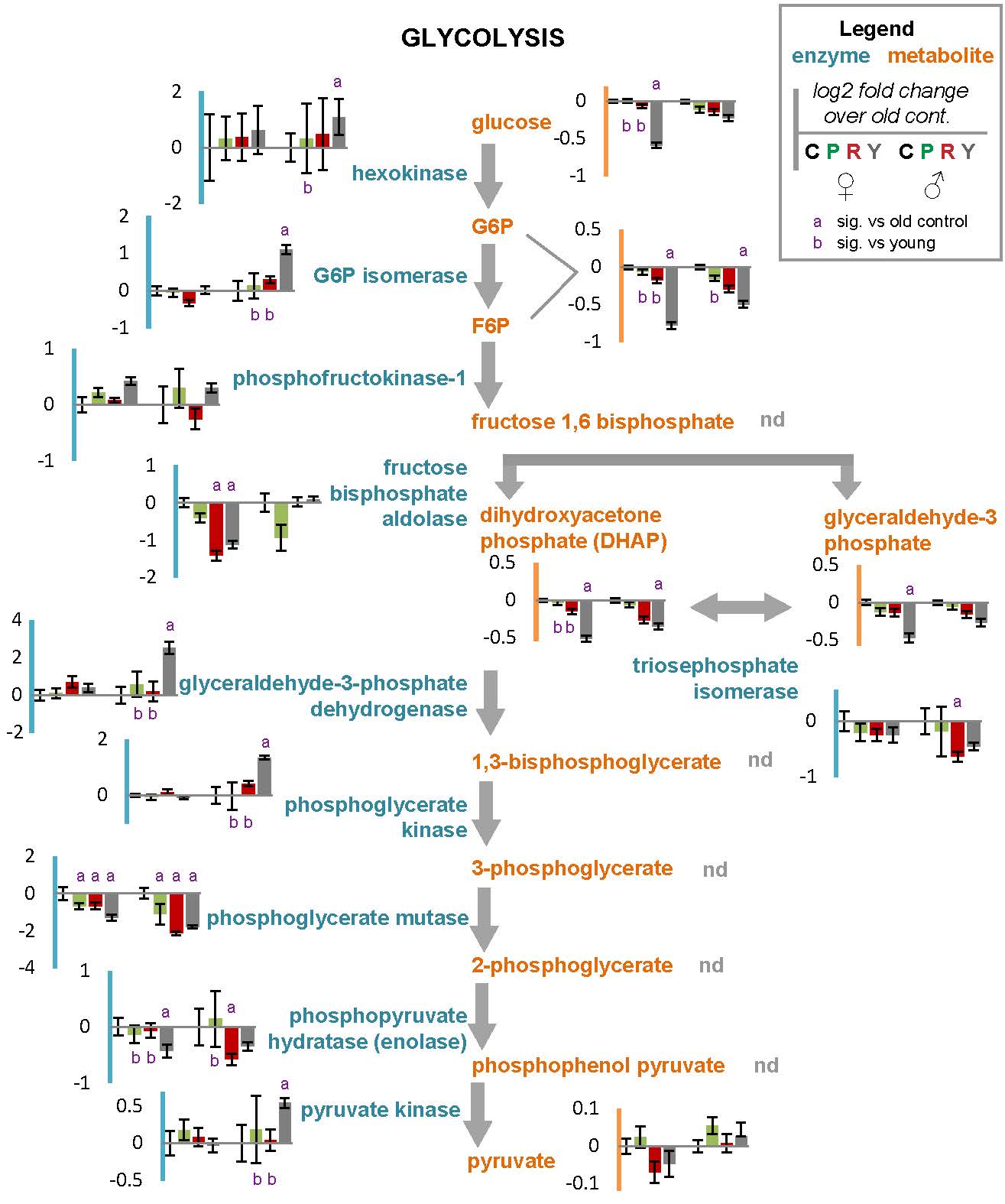


**Supporting Figure 5.** **Metabolomic and proteomic abundance changes in glycolysis.** This diagram shows the log2-fold-change from old control mice, in both sexes, in the enzymes (blue) and metabolites (orange) present in glycolysis. nd – no data. Statistical significance by one-way ANOVA followed by Tukey post-hoc test: group was significantly different compared to old control (a) or group was significantly different from young control (b) of the same sex. G6P and F6P were indistinguishable by metabolomics.

Supporting Figure 6. p16 mRNA and histology


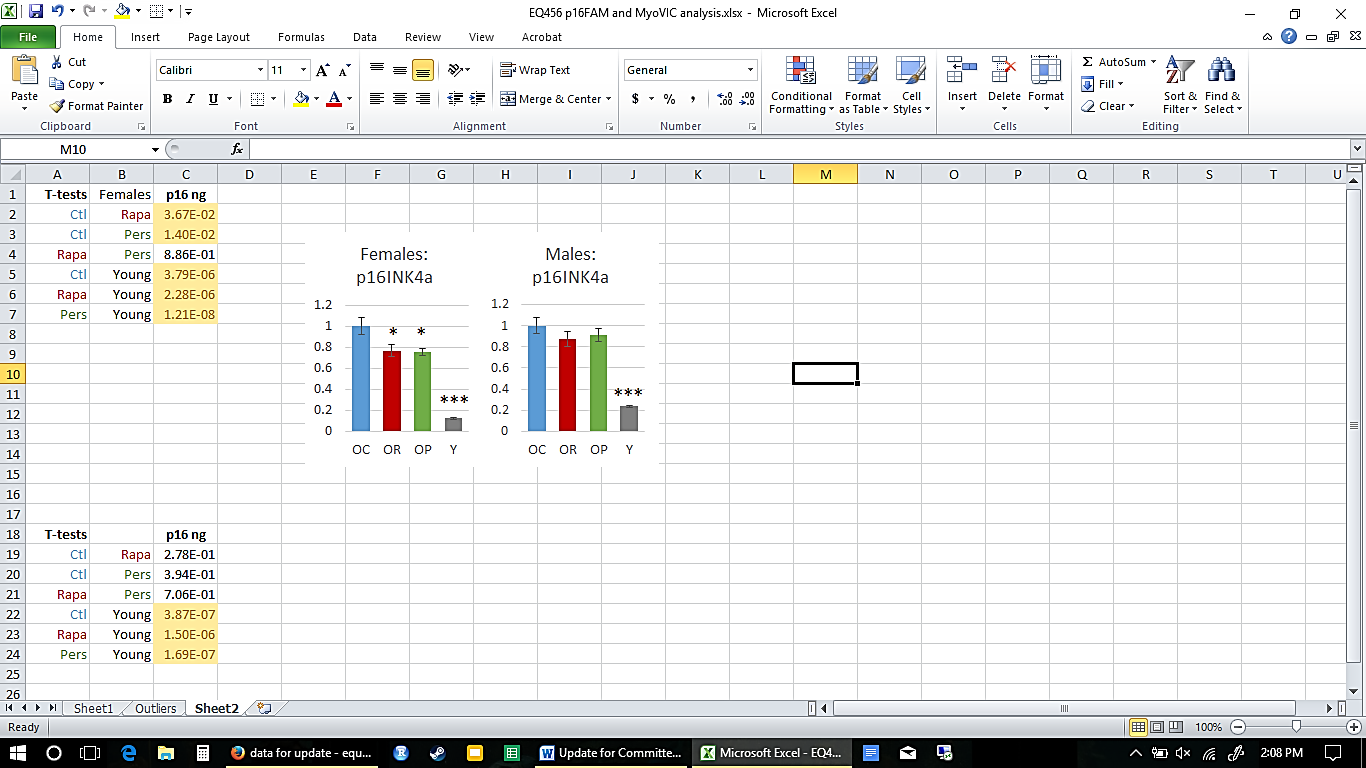
 **
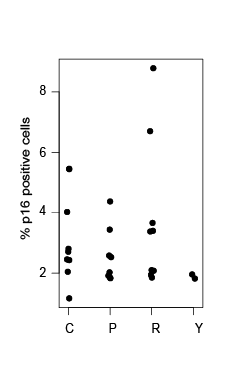
**

**Supporting Figure 6. p16INK4a mRNA quantities are increased with age in both sexes and persistently decline in females after rapamycin treatment.** Bar charts show relative quantities of mRNA (cDNA) for p16INK4a compared to old controls for each sex. N = 7~8 per group. Error bars are SEM. P-values from one-way ANOVA followed by Tukey post-hoc tests: * p<0.05, **p<0.01, *** p<0.001. OC – old control, OR – old rapa, OP – old persistence, Y – young. Percent p16 positive cells in male mice, by staining with anti-p16^INK4a^ antibody. No significant differences detected between groups except C vs Y by T-test. N = 8~9 per group, except young (n = 2).

**Cellular Senescence**

Senescence is an irreversible arrest of cell proliferation leading to radically altered cellular function and signaling. Selectively removing senescent cells results in longer lifespan and improved tissue function (Wang et al. 2017), including in the heart (Zhu et al. 2015). Rapamycin can inhibit some aspects of cell senescence in vitro (Wang et al. 2017), which lead us to question whether the senescent cell burden was reduced persistently in our rapamycin treated cohorts. Therefore, we investigated whether rapamycin alters the proportion of senescent cells using qPCR. The p16 tumor suppressor is a key mediator of senescence (Rayess et al. 2012). By qPCR, we found that expression of p16INK4a was increased with age, and reduced persistently with rapamycin in female, but not male, mice.
